# Social Self-Sorting of Quasi-Racemates: A Unique Approach for Dual-Pore Molecular Crystals

**DOI:** 10.1021/jacs.4c01654

**Published:** 2024-06-25

**Authors:** Momoka Kimoto, Shoichi Sugiyama, Keigo Kumano, Satoshi Inagaki, Suguru Ito

**Affiliations:** † Department of Chemistry and Life Science, Graduate School of Engineering Science, Yokohama National University, 79-5 Tokiwadai, Hodogaya-ku, Yokohama 240-8501, Japan; ‡ PRESTO, Japan Science and Technology Agency (JST), 4-1-8 Honcho, Kawaguchi, Saitama 332-0012, Japan

## Abstract

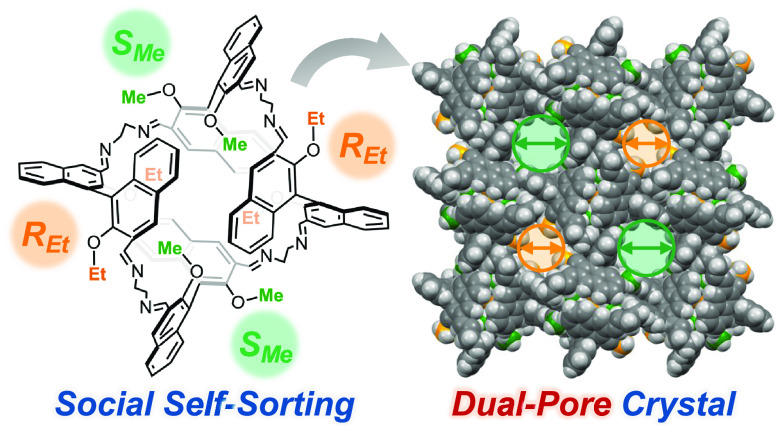

Despite recent advances in porous organic molecular crystals, the engineering of dual-pore systems within the intermolecular voids remains a significant challenge. In this study, we have achieved the crystallization-induced social self-sorting of “quasi-racemic” dialdehydes into a macrocyclic imine. X-ray crystallographic analysis unambiguously characterizes the resulting structure as incorporating two quasi-racemate pairs with four diamine molecules. Notably, different alkyl substituents on the quasi-racemates afford two types of one-dimensional pores within the macrocyclic imine crystal. The different adsorption properties of these pores were substantiated through adsorption experiments. An intriguing helical arrangement of guest molecules was observed within one of the pores. This study provides pioneering evidence that the social self-sorting of quasi-racemates offers a new methodology for creating dual-functional supramolecular materials.

Dynamic covalent bonds serve as powerful tools for the self-assembly of discrete supramolecular structures.^1^ Typically, a binary combination of precursors, each bearing complementary functional groups, is employed to construct a thermodynamically stable product. In contrast, multicomponent systems, which include at least two precursors having identical functional groups, remain relatively unexplored.^2−5^ To achieve “social self-sorting” of different precursors with the same functional groups into a unified supramolecular structure,^2,3^ strategic approaches are required to prevent random incorporation and “narcissistic self-sorting”,^4,5^ where each type of precursor assembles into independent structures. One approach is to restrict the orientation and number of functional groups.^2^ Another noteworthy approach is chiral self-sorting, which relies on the complementarity of chirality.^3^ When a racemic precursor is used, both enantiomers are frequently incorporated into a single structure (Figure 1a). As a limited yet valuable instance, chiral complementarity also allows for the social self-sorting of “quasi-racemic” precursors (Figure 1b),^6,7^ where enantiomers have almost identical structures with slight variations in substituents. The previous report^6^ on quasi-racemic systems has focused on the construction of socially self-sorted structures, and the utilization of different substituents of quasi-racemates in functional materials remains unexplored.

**Figure 1:**
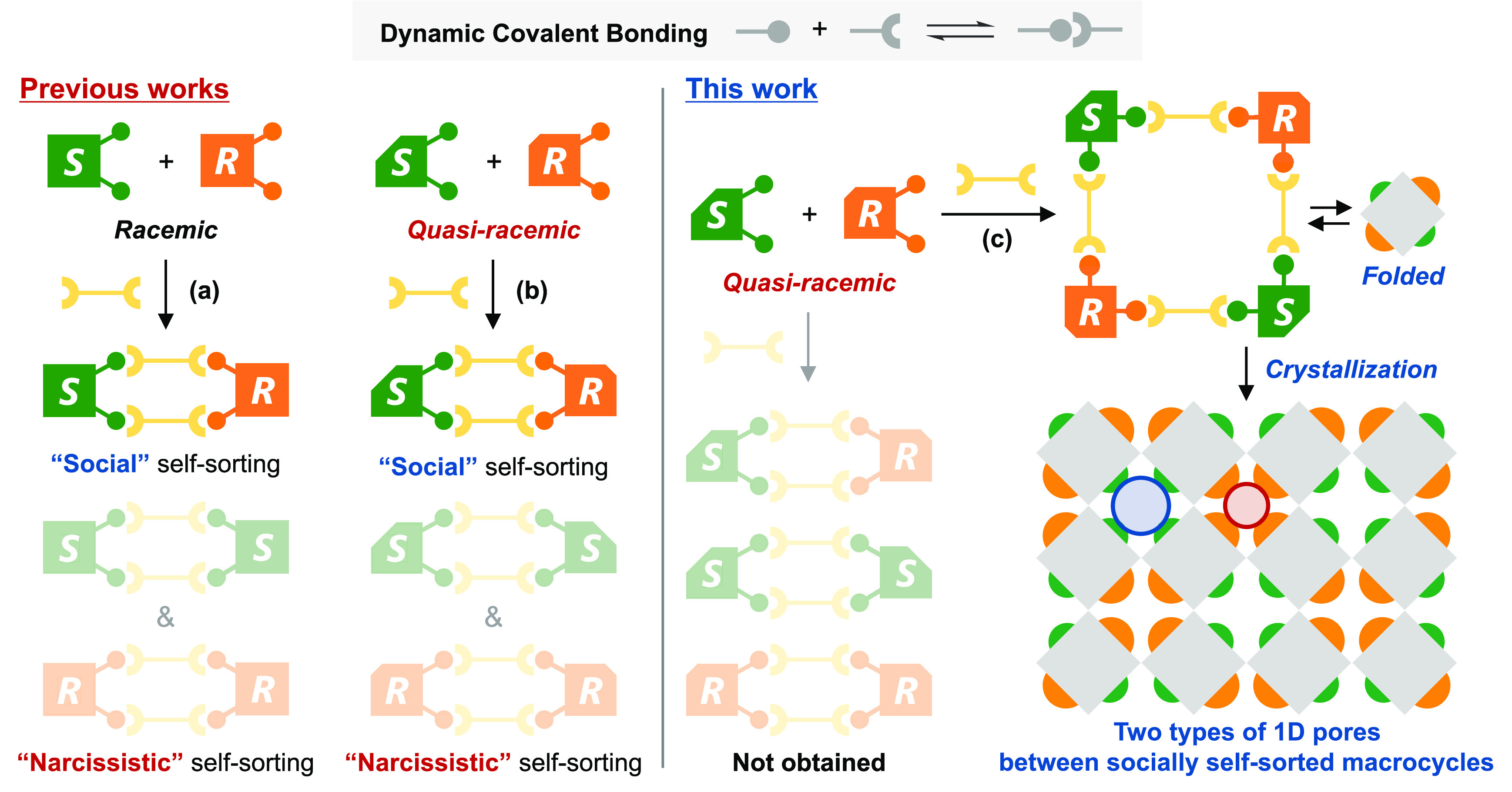
Comparison of previous works and this work in chiral social self-sorting. (a) Social self-sorting of racemic precursors. (b) Social self-sorting of quasi-racemic precursors. (c) Social self-sorting of quasi-racemic precursors followed by crystallization into porous molecular crystals with two types of 1D pores.

Discrete organic macrocycles and cages formed by dynamic covalent bonding occasionally crystallize into porous molecular crystals, which show great promise in applications such as molecular separations, gas storage, and catalysis.^1e,8^ In contrast to covalent organic frameworks (COFs),^9^ the precise structural determination of pores can be readily achieved for molecular crystals through single-crystal X-ray diffraction analysis.^10^ One-dimensional (1D) channels are formed by the internal spaces within the macrocycles,^11^ while cage crystals typically have two-dimensional and three-dimensional porosity, arising from cavities within the molecules and voids between them.^12,13^ These porous structures are suitable for the adsorption and separation of gas molecules and vapors of liquid organic compounds. Despite the recent surge in extensive studies on porous crystalline macrocycles and cages built via dynamic covalent bonding, their structures are limited to those arising from narcissistic self-sorting or constructed using racemic precursors.

Designing porous materials with dual-pore systems presents a complex task, yet such materials are highly valuable due to their advanced functionalities. Because each pore can be functionalized distinctly, dual-pore materials enable simultaneous multiple functions or specific designs for complex applications. In the realm of COFs, there has been a recent rapid increase in examples where the size of pores is tailored to create heterogeneous pore environments.^14^ The latest studies highlight the emergence of dual-pore COFs demonstrating sophisticated functions that are unattainable by single-pore systems.^15^ However, achieving dual-pore systems in molecular crystals remains a challenging endeavor,^16,17^ particularly in the context of creating two different types of 1D pores. As single-crystal X-ray analysis allows precise determination of molecular crystals, developing new methods for dual-pore molecular crystals is expected to produce advanced materials with precisely controlled pore structures.

Herein, we have achieved the social self-sorting of two pairs of quasi-racemic dialdehydes, together with four diamine molecules, into a macrocyclic imine (Figure 1c). In sharp contrast to the previous study, where the self-sorted arrangement of quasi-racemic precursors was identical to that of racemic precursors,^6^ the present macrocyclic structure is unattainable with racemic precursors. Most remarkably, the crystals of the folded macrocycle contain two distinct 1D pores surrounded by either methyl or ethyl groups of the quasi-racemic components. Experimental evidence has confirmed that these dual pores exhibit different adsorption properties. To the best of our knowledge, this is the first dual-pore molecular crystal formed by socially self-sorted macrocycles.^18^

A combination of dialdehydes (*S*)-**1a**^19^ and (*R*)-**1b**^20^ having methyl and ethyl groups, respectively, was selected as quasi-racemic precursors with sterically different substituents (Figure 2a). The self-sorting of the quasi-racemic (*S*)-**1a** and (*R*)-**1b** into a macrocyclic imine was examined through dynamic imine bond formation using ethylenediamine (**2**) (Tables S1 and S2, Figures S1 and S2). When (*S*)-**1a**, (*R*)-**1b**, and **2** were stirred in toluene (100 mM) at room temperature for 2 d, almost pure macrocyclic imine was obtained as white precipitates in 81% yield (Figure 2a). The molecular structure was identified as (*S*,*R*,*S*,*R*)-**3** by ^1^H NMR and ESI-HRMS spectra. Based on the ^1^H NMR spectrum, the quasi-racemic (*S*)-**1a** and (*R*)-**1b** are incorporated in the macrocyclic imine in a ratio of 1:1 (Figure 2b). Furthermore, ESI-HRMS analysis revealed that (*S*,*R*,*S*,*R*)-**3** is a [2_*S*_ + 2_*R*_ + 4]-type macrocyclic imine incorporating two pairs of quasi-racemic molecules and four diamine molecules (Figures S3–S6). For the [2_*S*_ + 2_*R*_ + 4]-type macrocycle, two isomers, *SRSR* and *SSRR*, are possible (Figure 2a). Since the ^1^H NMR spectrum indicates the formation of a symmetrical structure (Figure 2b), **3** is the *SRSR* isomer.

**Figure 2:**
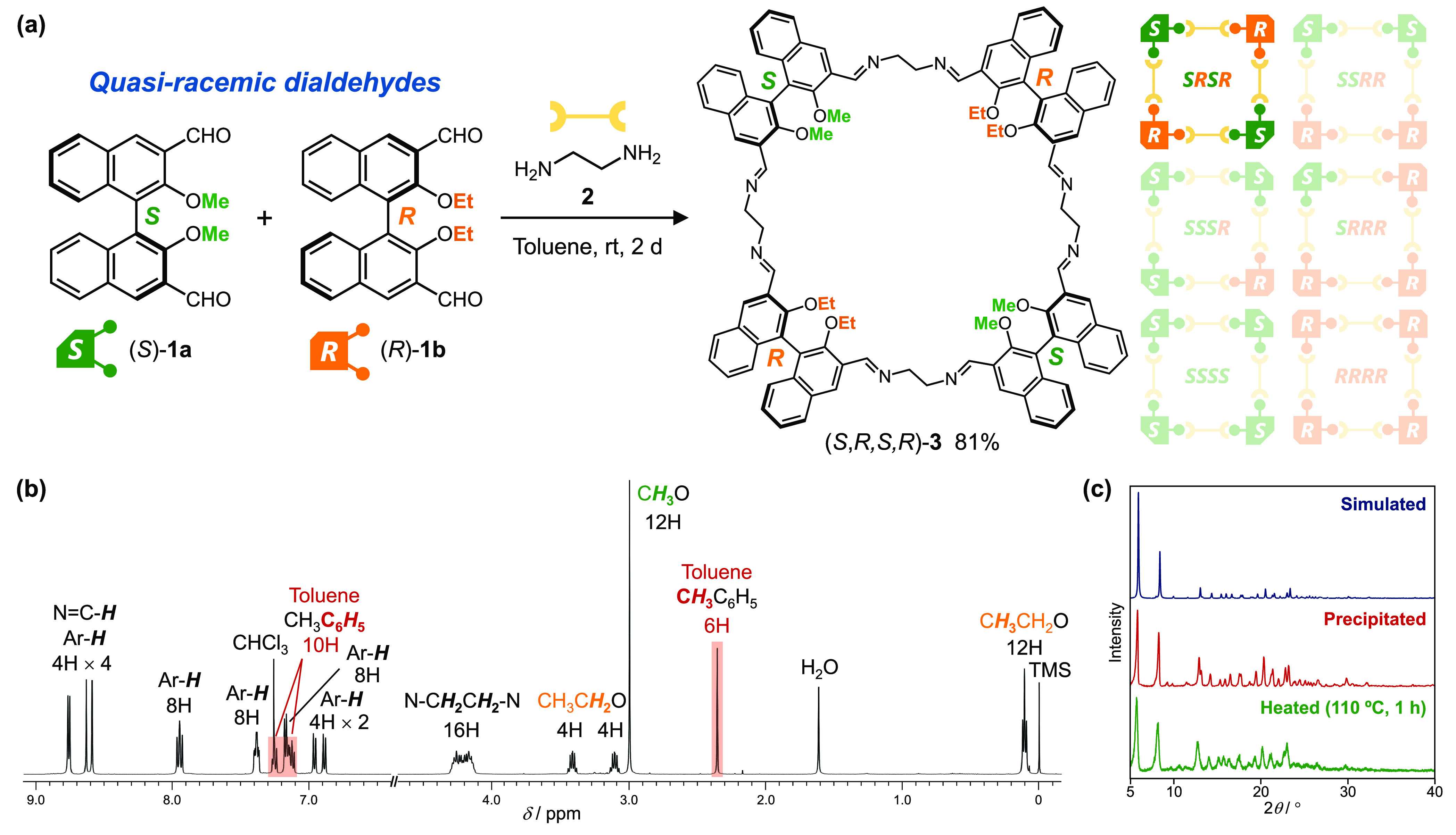
(a) Self-assembly of [2_*S*_ + 2_*R*_ + 4] macrocyclic imine (*S*,*R*,*S*,*R*)-**3** from (*S*)-**1a**, (*R*)-**1b**, and **2**. (b) ^1^H NMR (500 MHz) spectrum of (*S*,*R*,*S*,*R*)-**3**·toluene in CDCl_3_ at room temperature. (c) PXRD patterns of (*S*,*R*,*S*,*R*)-**3**: simulated from the single-crystal structure (blue), precipitates (red), and after heating the precipitates at 110 °C for 1 h (green).

The selective formation of (*S*,*R*,*S*,*R*)-**3** is attributed to the crystallization-induced self-assembly.^21^ Powder X-ray diffraction (PXRD) analysis of the precipitate showed sharp diffraction patterns, indicating that (*S*,*R*,*S*,*R*)-**3** was obtained as a crystalline powder (Figure 2c). The crystalline precipitate of (*S*,*R*,*S*,*R*)-**3** contained toluene molecules at a molar ratio of 1:2 (Figure 2b). Temporal ^1^H NMR analyses of the reaction confirmed the consistent presence of oligomeric mixtures in solution, while the precipitate was predominantly composed of (*S*,*R*,*S*,*R*)-**3** from the initial stages (Figures S9 and S10). Accordingly, initial crystallization of (*S*,*R*,*S*,*R*)-**3**·toluene from the reaction mixture should induce an equilibrium bias that favors the formation of (*S*,*R*,*S*,*R*)-**3**, leading to the selective growth of crystalline (*S*,*R*,*S*,*R*)-**3**·toluene.

Single-crystal X-ray diffraction analysis confirmed the molecular structure of (*S*,*R*,*S*,*R*)-**3** (Figures 3 and S11–S14). The simulated PXRD pattern of the single crystal was closely matched that of the powder precipitated from the reaction mixture (Figure 2c). As identified by ^1^H NMR and MS spectra, (*S*,*R*,*S*,*R*)-**3** is composed of two pairs of quasi-racemic dialdehydes in the order of *SRSR*. Each molecule in the crystal exhibited a folded conformation (Figure 3a). Of the two alkoxy substituents on each binaphthyl moiety, one oriented toward the inner side of the macrocyclic ring, while the other faced the outer side. Accordingly, the interior of the macrocycle, surrounded by four naphthalene rings, is filled with two methoxy groups and two ethoxy groups (Figure S12). In self-assembly utilizing dynamic covalent bonding, template molecules often guide supramolecular host construction by occupying their internal spaces.^22^ In the present system, the alkoxy groups are analogous to templates, which accounts for the selective formation of (*S*,*R*,*S*,*R*)-**3**.

**Figure 3:**
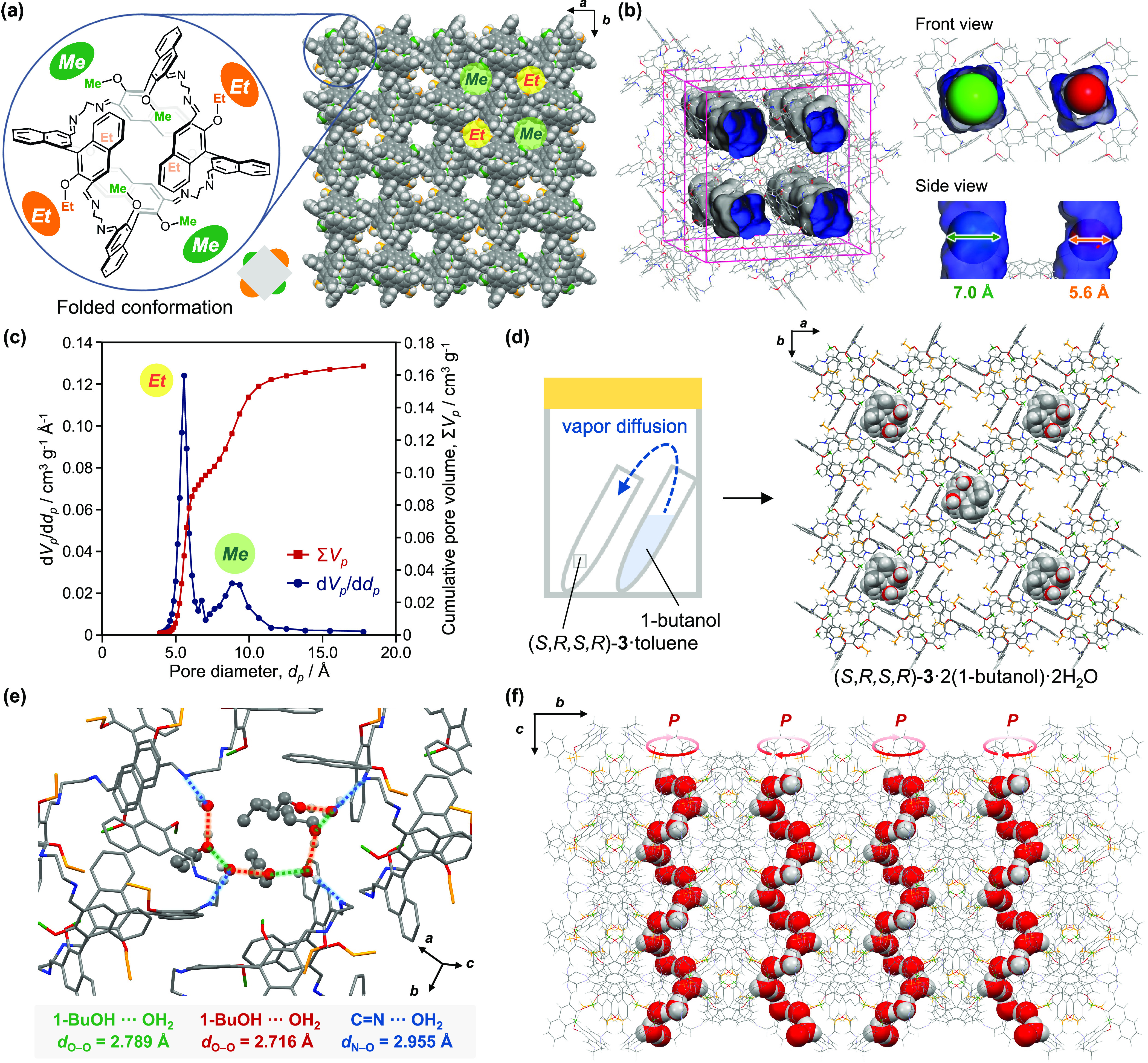
(a) Folded molecular structure and packing structure of (*S*,*R*,*S*,*R*)-**3** viewed from *c*-axis. (b) Estimation of pore volumes and diameters of crystalline (*S*,*R*,*S*,*R*)-**3** by calculating via a rolling-ball algorithm developed by Connolly. (c) SF-plot estimated from the nitrogen adsorption isotherm (77 K) of (*S*,*R*,*S*,*R*)-**3**. (d) Vapor diffusion of 1-butanol into crystalline (*S*,*R*,*S*,*R*)-**3**·toluene. Included 1-butanol and H_2_O molecules are depicted in CPK model. (e) Hydrogen bonding network formed by (*S*,*R*,*S*,*R*)-**3**, 1-butanol, and H_2_O. (f) Helically arranged extended hydrogen bonding between 1-butanol and H_2_O in crystalline (*S*,*R*,*S*,*R*)-**3** viewed from *a*-axis. The OH groups of 1-butanol and H_2_O molecules are depicted in CPK model.

Both methyl and ethyl groups are indispensable for the construction of [2_*S*_ + 2_*R*_ + 4]-type macrocyclic imine (*S*,*R*,*S*,*R*)-**3**. When *rac*-**1a** and *rac*-**1b** were independently stirred with **2** under the same reaction conditions as the self-assembly of (*S*,*R*,*S*,*R*)-**3**, [1_*S*_ + 1_*R*_ + 2]-type (*S*,*R*)-**4** incorporating one molecule each of (*S*)-**1a** and (*R*)-**1a**, and oligomeric imine mixtures were obtained, respectively (Table S3 and Figures S7, S8, S15, and S16). Another important factor is the complemental chirality of quasi-racemates, which facilitate the social self-sorting of two alkoxy groups. Indeed, (*S*)-**1a** and (*S*)-**1b** yielded a complex mixture (Figure S17), demonstrating the difficulty in sorting sterically similar components that have the same chirality.

Remarkably, two distinct 1D micropores are confirmed in the crystal of (*S*,*R*,*S*,*R*)-**3** when the packing structure is viewed along the *c*-axis direction (Figure 3). The molecules exhibit a repeating arrangement characterized by a 90° rotation between adjacent molecules in both the *a*- and *b*-axis directions, and they stack in the *c*-axis direction without any displacement (Figures 3a, S13, and S14). Since methyl and ethyl groups are present around the periphery of each molecule at ca. 90° intervals, the two distinct pores with different sizes are formed within the intermolecular spaces of molecules arranged in the *ab*-plane: larger pores surrounded by methyl groups and smaller pores surrounded by ethyl groups. These pores should contain toluene molecules, although the electron densities in the pores could not be assigned to the highly disordered toluene molecules and were eliminated using the SQUEEZE method.^23^ By calculating the Connolly surface (Connolly radius = 1.8 Å), the pore volumes were estimated to be 0.1155 cm^3^·g^–1^ and 0.0865 cm^3^·g^–1^ for pores surrounded by methyl and ethyl groups, respectively (Figure 3b). The pore diameters were also estimated to be 7.0 and 5.6 Å (Figure 3b).

Experimental confirmation of the two micropores in crystalline (*S*,*R*,*S*,*R*)-**3** was achieved through nitrogen adsorption measurements. Toluene molecules in the micropores could be removed by heating crystalline precipitate (*S*,*R*,*S*,*R*)-**3**·toluene at 110 °C for 1 h (Figure S18). The PXRD analysis of the heated sample confirmed the retention of the initial crystal structure (Figure 2c). The nitrogen adsorption experiment was carried out at 77 K for the toluene-free crystalline (*S*,*R*,*S*,*R*)-**3**. A rapid uptake of nitrogen molecules was observed in the relative pressure range *P*/*P*_0_ = 0–0.01 to afford a type I isotherm (Figure S19a). The Brunauer–Emmett–Teller (BET) surface area^24^ was determined to be 449 m^2^·g^–1^ (Figure S19b). In the isotherm plotted with a logarithmic scale on the horizontal axis, two stages of adsorption were confirmed, which should originate from the two types of micropores with different sizes (Figure S19c). Based on the α_s_-plot analysis,^24,25^ the micropore volumes were determined to be 0.0906 cm^3^·g^–1^ and 0.0801 cm^3^·g^–1^ for larger and smaller pores, respectively (Figure S19d). In addition, the micropore diameters were calculated by Saito and Foley method (SF-plot)^24,26^ to be 9.4 and 5.6 Å (Figure 3c), which should correspond to the methyl- and ethyl-surrounded pores, respectively, and are comparable to the values simulated from the single-crystal X-ray structure (Figure 3b).

Distinct adsorption characteristics were confirmed for the two 1D micropores of crystalline (*S*,*R*,*S*,*R*)-**3**. Single crystals of (*S*,*R*,*S*,*R*)-**3**·toluene were exposed to the vapor of various alcohols (Figures S20 and S21). Only when exposed to 1-butanol vapor, the adsorption of 1-butanol molecules could be confirmed by X-ray crystallography (Figures 3d–f and S22). For one molecule of (*S*,*R*,*S*,*R*)-**3**, two molecules of 1-butanol were included in the larger pore together with two molecules of H_2_O. The H_2_O molecules are likely derived from the moisture either present in the 1-butanol or in the atmosphere. Each H_2_O molecule formed hydrogen bonds with the imine moiety of (*S*,*R*,*S*,*R*)-**3** and adjacent two 1-butanol molecules (Figures 3e and S23). Interestingly, 1-butanol and H_2_O molecules, forming the extended hydrogen bonds, were arranged in a single-handed helical structure with *P*-helicity (Figure 3f and Movie S1). This structure reflects the chiral arrangement of the imine moiety of (*S*,*R*,*S*,*R*)-**3** exposed inside the larger pore. The ^1^H NMR analysis of (*S*,*R*,*S*,*R*)-**3**·1-butanol·H_2_O indicated the inclusion of three 1-butanol molecules (Figure S24), suggesting the presence of 1-butanol molecules in the smaller pores. However, the electron densities present in the smaller pores could not be assigned to 1-butanol due to severe disorder, indicating a marked difference in molecular fluidity within the two types of micropores.

In summary, the socially self-sorted macrocyclic imine (*S*,*R*,*S*,*R*)-**3** was selectively obtained from quasi-racemic dialdehydes (*S*)-**1a** and (*R*)-**1b** by crystallization-induced self-assembly using diamine **2**. Most impressively, the crystals composed of (*S*,*R*,*S*,*R*)-**3** feature two types of 1D pores with different sizes surrounded by methyl and ethyl groups of the socially self-sorted quasi-racemic components. The distinct adsorption properties of these pores were demonstrated by the helical arrangement of 1-butanol and H_2_O molecules into the larger pores mediated by extended hydrogen bonding. This study emphasizes the exceptional utility of quasi-racemates in constructing socially self-sorted supramolecular structures with two distinct functionalities. Furthermore, the methodology sets the stage for the generation of a novel class of dual-pore molecular crystals. Future investigations are expected to advance the utility of socially self-sorted molecular crystals in the precise separation of different guest molecules and the creation of multifunctional supramolecular materials.

## Supporting Information Available

Experimental procedures, screening of reaction conditions, HRMS-ESI spectra, NMR spectra, crystallographic data, and adsorption experiments (PDF)
Movie S1, showing the crystal structure displaying the helical arrangements of 1-butanol and H_2_O molecules (MP4)
