# Supplementary material for: Social Self-Sorting of Quasi-Racemates: A Unique Approach for Dual-Pore Molecular Crystals
Source: J Am Chem Soc. 2024 Jun 25;146(26):17559–65. doi: 10.1021/jacs.4c01654 (PMC11229008; doi:10.1021/jacs.4c01654)

Social Self-Sorting of Quasi-Racemates: A Unique Approach for Dual-Pore Molecular Crystals

Momoka Kimoto,† Shoichi Sugiyama,† Keigo Kumano,† Satoshi Inagaki,† and Suguru Ito*,†,‡

†*Department of Chemistry and Life Science, Graduate School of Engineering Science,*

Yokohama National University

79-5 Tokiwadai, Hodogaya-ku, Yokohama 240-8501, Japan

‡*PRESTO, Japan Science and Technology Agency (JST)*

4-1-8 Honcho, Kawaguchi, Saitama 332-0012, Japan

*E-mail: suguru-ito@ynu.ac.jp

Table of contents

1. General…….. S2

2. Experimental Procedure and Characterization of New Compounds.. S3

3. Screening of Reaction Conditions..………….. S5

4. HRMS-ESI Analyses.. S9

5. Temporal ^{1}H NMR Spectra of Supernatants and Precipitates…... S12

6. Single-Crystal X-ray Diffraction Analyses………………………………………………. S15

7. Dynamic Imine Bonding Reactions of Racemic Precursors………………………………….. S19

8. Nitrogen Adsorption Isotherm Measurement of (*S*,*R*,*S*,*R*)-**3**…………………………………….. S23

9. Adsorption of Alcohol Vapors………………….. S25

10. References …………………..………………………………………………….................. S28

^{1}H and ^{13}C NMR spectra...…………………………………..…………………….................. S29

1. General

All air-sensitive experiments were carried out under an atmosphere of argon unless otherwise noted. FTIR spectra were recorded on a JASCO FT/IR 6200 spectrometer. ^{1}H and ^{13}C NMR spectra were recorded on a Bruker DRX-500 spectrometer or a JEOL ECA-500 spectrometer using tetramethylsilane (^{1}H NMR: 0.00 ppm) or solvent residual signal [^{13}C NMR: $CDCl_3$ (77.0 ppm)] as an internal standard. Optical rotations were measured on an Anton Paar MCP 150 polarimeter. Melting points were determined on a Stuart melting point apparatus SMP3 and are uncorrected. High-resolution electrospray ionization mass spectra (HRMS-ESI) were recorded on a Hitachi Nano Frontier LD spectrometer. Powder X-ray diffraction (PXRD) measurements were performed on a Rigaku SmartLab system using CuKα radiation. Micropore volumes and micropore diameters within the single-crystal structure were estimated via a rolling-ball algorithm developed by Connolly (Connolly radius is 1.8 Å) using Materials Studio software. 2,2′-Dimethoxy-1,1′-binaphthalene-3,3′-dicarbaldehyde (**1a**)[1] and 2,2′-diethoxy-1,1′-binaphthalene-3,3′-dicarbaldehyde (**1b**)[2] were synthesized according to the literature procedure. Toluene was distilled according to the usual procedures and stored over molecular sieves. Other reagents and solvents were commercially available and were used as received.

2. Experimental Procedure and Characterization of New Compounds

2.1. Social self-sorting of (*S*)-**1a** and (*R*)-**1b** into (*S*,*R*,*S*,*R*)-**3**

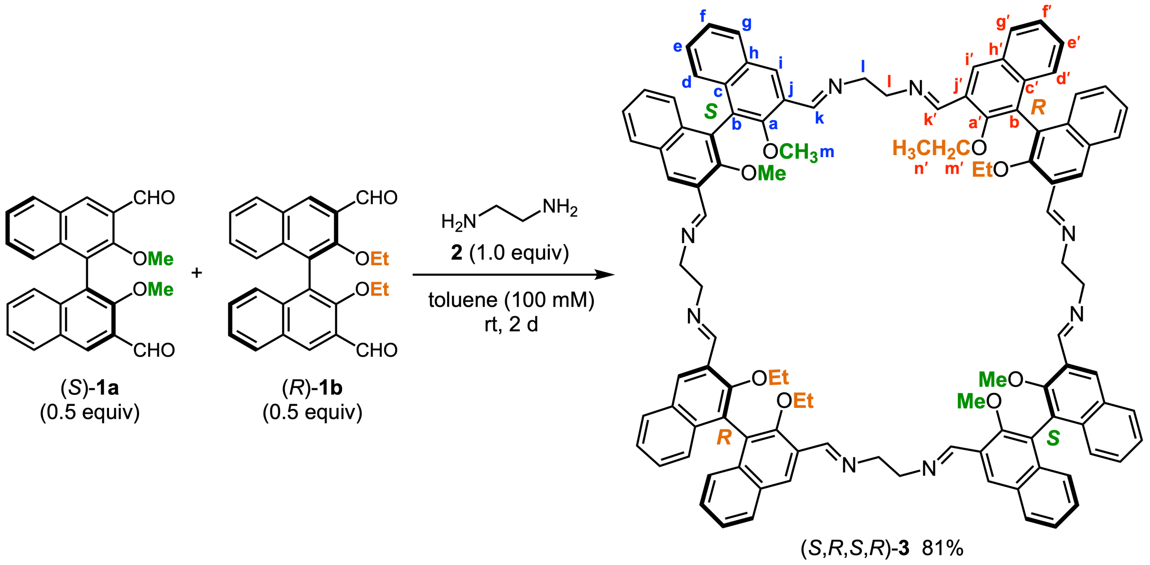

A mixture of (*S*)-2,2′-dimethoxy-1,1′-binaphthalene-3,3′-dicarbaldehyde (**1a**) (18.5 mg, 0.050 mmol), (*R*)-2,2′-diethoxy-1,1′-binaphthalene-3,3′-dicarbaldehyde (**1b**) (19.9 mg, 0.050 mmol), and ethylenediamine (**2**) (7.0 µL, 0.100 mmol) in toluene (1.0 mL, 100 mM) was stirred at room temperature for 2 d. After filtration of the resulting suspension under reduced pressure, (*S*,*R*,*S*,*R*)-**3**·toluene was obtained as a white powder (35.1 mg, 81% yield).

White solid; Mp. 238.0–238.4 °C; $[\alpha]_D^{25}$ −29.0 (*c* 1.0, $CHCl_3$); IR (KBr): ν_{max} 3061, 2889, 2837, 1619, 1496, 1351, 1239, 1105, 1033, 744 cm^{-1}; ^1H NMR (500 MHz, $CDCl_3$): δ (ppm) 8.77 (s, 4H, $H_{k'}$), 8.75 (s, 4H, H_k), 8.63 (s, 4H, H_i), 8.59 (s, 4H, $H_{i'}$), 7.98–7.94 (m, 8H, $H_{g,g'}$), 7.41–7.37 (m, 8H, $H_{f,f'}$), 7.28–7.24 (m, 4H, tol), 7.19–7.11 (m, 8H, $H_{e',e}$ + 6H, tol), 6.96 (d, J = 8.6 Hz, 4H, $H_{d'}$), 6.89 (d, J = 8.6 Hz, 4H, H_d), 4.29–4.14 (m, 16H, $H_{l,l'}$), 3.45–3.38 (m, 4H, $H_{m'}$), 3.14–3.06 (m, 4H, $H_{m'}$), 2.99 (s, 12H, H_m), 2.36 (s, 3H, tol) 0.10 (t, J = 7.0 Hz, 12H, $H_{n'}$); ^{13}C NMR (126 MHz, $CDCl_3$, −20 °C): 159.9 ($C_{k'}$), 159.3 (C_k), 154.8 (C_a), 154.5 ($C_{a'}$), 137.9 (tol), 134.8 ($C_{c,c'}$), 130.1 (C_h), 129.9 ($C_{h'}$), 129.3 (C_g), 129.2 ($C_{g'}$), 129.0 (tol, 2C), 128.5 ($C_{j'}$), 128.19 (C_j), 128.15 (tol, 2C), 128.13 (C_i), 127.9 ($C_{i'}$), 127.6 ($C_{e'}$), 127.4 (C_e), 125.3 (C_f), 125.20 (tol), 125.17 ($C_{f'}$), 125.1 ($C_{d'}$), 125.0 (C_d), 124.9 (C_b), 124.4 (C_b), 70.9 ($C_{m'}$), 62.32 (C_l), 62.25 (C_l), 62.0 (C_m), 21.5 (tol), 15.0 ($C_{n'}$); HRMS-ESI (*m*/*z*): $[M + H]^+$ calcd for $C_{108}H_{97}N_8O_8$, 1633.7424; Found, 1633.7423. $[M + 2H]^{2+}$ calcd for $C_{108}H_{98}N_8O_8$, 817.3748; Found, 817.3752. $[M + 3H]^{3+}$ calcd for $C_{108}H_{99}N_8O_8$, 545.2523; Found, 545.2523.

2.2. Social self-sorting of (*S*)-**1a** and (*R*)-**1a** into (*S*,*R*)-**4**

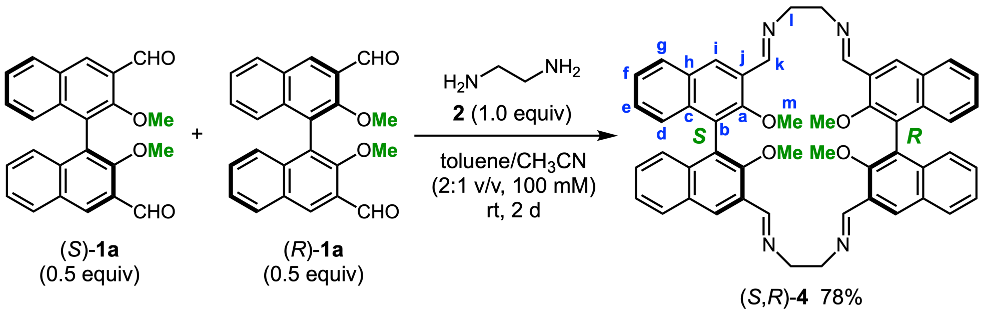

(*S*)-**1a** (0.5 equiv) (*R*)-**1a** (0.5 equiv) (*S*,*R*)-**4** 78%

A mixture of (*S*)-**1a** (9.3 mg, 0.025 mmol), (*R*)-**1a** (9.3 mg, 0.025 mmol), and **2** (3.5 μL, 0.050 mmol) in toluene/CH_3CN (2:1 v/v, 0.50 mL, 100 mM) was stirred at room temperature for 2 d. After filtration of the resulting suspension under reduced pressure, (*S*,*R*)-**4** was obtained as a white powder (15.4 mg, 78% yield).

White solid; Mp. 299.2–299.5 °C; IR (KBr): ν_{max} 3057, 2906, 2840, 1616, 1456, 1355, 1240, 1107, 1006, 901, 746 cm^{-1}; ^1H NMR (500 MHz, $CDCl_3$): δ (ppm) 8.81 (s, 4H, H_k), 8.42 (s, 4H, H_i), 7.92 (d, J = 8.1 Hz, 4H, H_g), 7.39–7.36 (m, 4H, H_f), 7.26–7.23 (m, 4H, H_e), 7.16 (d, J = 8.6 Hz, 4H, H_d), 4.39–4.34 (m, 4H, H_l), 4.22–4.16 (m, 4H, H_l), 3.23 (s, 12H, H_m); ^{13}C NMR (126 MHz, $CDCl_3$): δ (ppm) 159.8 (C_k), 155.1 (C_a), 135.2 (C_c), 130.18 (C_i), 130.14 (C_h), 128.91 (C_g), 128.84 (C_j), 127.4 (C_e), 125.7 (C_d), 125.4 (C_b), 125.1 (C_f), 61.6 (C_m), 60.9 (C_l); HRMS-ESI (m/z): $[M + H]^+$ calcd for $C_{52}H_{45}N_4O_4$, 789.3435; Found, 789.3436.

3. Screening of Reaction Conditions

3.1. Screening of solvent

The self-assembly of a macrocyclic imine incorporating the quasi-racemic dialdehydes was examined through dynamic imine bond formation using (*S*)-**1a**, (*R*)-**1b**, and ethylenediamine (**2**) (Table S1). Specifically, (*S*)-**1a**, (*R*)-**1b**, and **2** were stirred in various solvents (71 mM) at room temperature for 2 d (entries 1–8). When stirred in THF, ethyl acetate, ethanol, acetonitrile, and toluene, white solids were precipitated and collected by filtration under reduced pressure (entries 1–5). In the ^{1}H NMR spectra of the precipitates, (*S*,*R*,*S*,*R*)-**3** was observed along with broad signals from the mixture of oligomeric imines (Figure S1). The highest ratio of (*S*,*R*,*S*,*R*)-**3** over other oligomers was confirmed in the precipitates obtained from toluene [(*S*,*R*,*S*,*R*)-**3**/others = 1:0.2], and the NMR yield of (*S*,*R*,*S*,*R*)-**3** was 36% (entry 5).

Table S1. Screening of the reaction solvents in the self-sorting of (*S*)-**1a** and (*R*)-**1b** into (*S*,*R*,*S*,*R*)-**3**.[a]

Entry	Solvent	(*S*,*R*,*S*,*R*)-**3**/others[b,c]	NMR yield (%)[c]
1	THF	1:42	2
2	EtOAc	1:18	trace
3	EtOH	1:1.4	27
4	CH_3CN	1:1.4	29
**5**	**Toluene**	**1:0.2**	**36**
6	MeOH	Not precipitated	–
7	$CHCl_3$	Not precipitated	–
8	Et_2O	Not precipitated	trace

[a] (*S*)-**1a**, (*R*)-**1b**, and **2** were mixed in solvent (71 mM) at room temperature for 2 d. [b] Other macrocycles and oligomers. [c] Determined by ^{1}H NMR analysis.

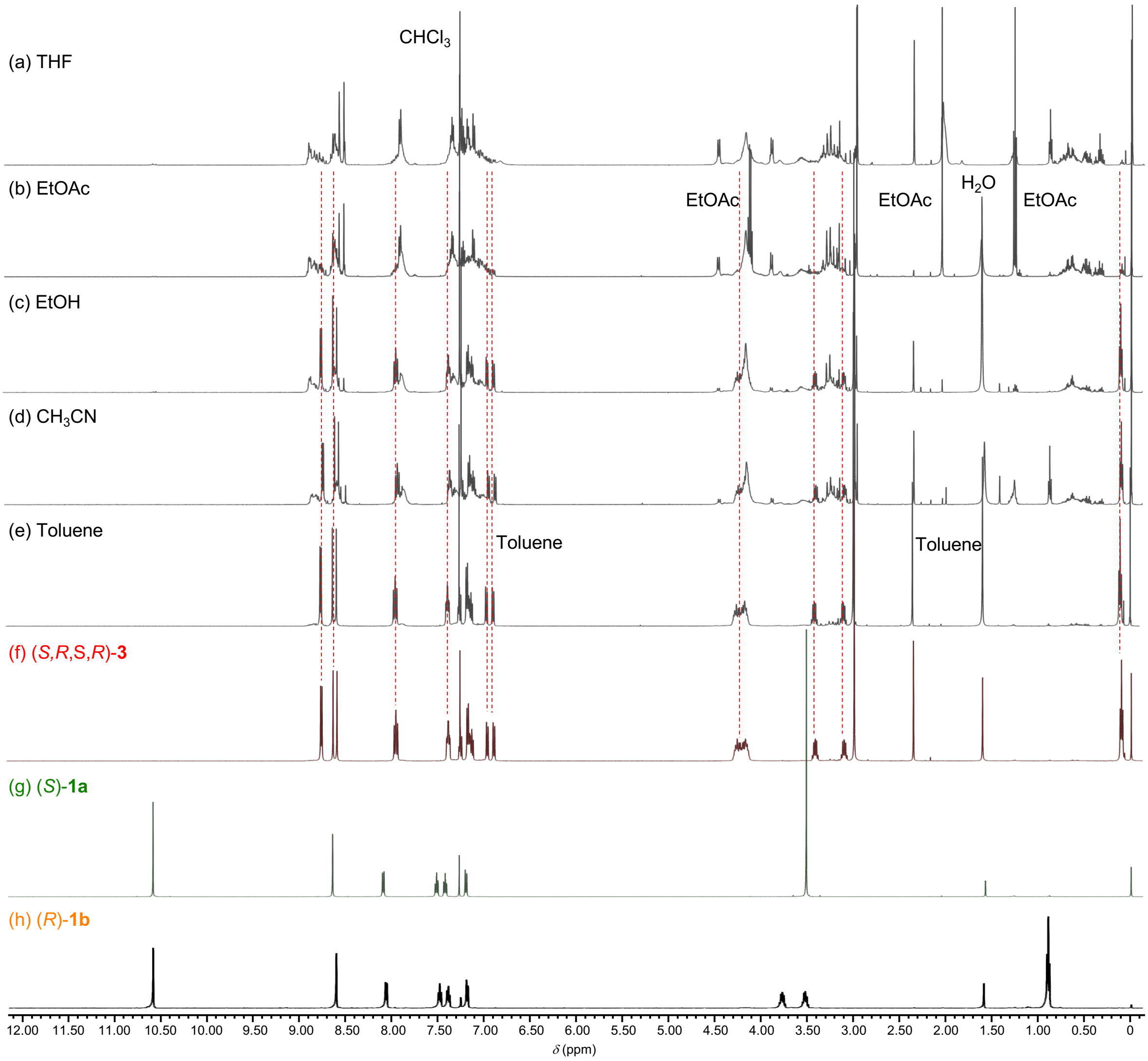

Figure S1. ^{1}H NMR spectra (500 MHz, in $CDCl_3$, rt) of macrocyclic and oligomeric imines precipitated from (a) THF (Table S1, entry 1), (b) EtOAc (Table S1, entry 2), (c) EtOH (Table S1, entry 3), (d) CH_3CN (Table S1, entry 4), and (e) toluene (Table S1, entry 5). ^{1}H NMR spectra (500 MHz, in $CDCl_3$, rt) of (f) (*S*,*R*,*S*,*R*)-**3**, (g) (*S*)-**1a**, and (h) (*R*)-**1b**.

3.2. Screening of reaction conditions in toluene

After screening the reaction conditions using toluene (Table S2 and Figure S2), both the selectivity and yield in the self-sorting of (*S*)-**1a**, (*R*)-**1b**, and **2** were significantly improved when the concentration was set at 100 mM, and almost pure macrocyclic imine (*S*,*R*,*S*,*R*)-**3** was obtained in 81% yield [entry 2 and Figure S2a: (*S*,*R*,*S*,*R*)-**3**/others = 1:<0.05].

Table S2. Screening of the reaction conditions in the self-sorting of (*S*)-**1a** and (*R*)-**1b** into (*S*,*R*,*S*,*R*)-**3** in toluene.[a]

Entry	Concentration (mM)	Temperature (°C)	(*S*,*R*,*S*,*R*)-**3**/others[b,c]	NMR yield (%)[c]
1	10	rt	Not precipitated	-
**2**	**100**	**rt**	**1:<0.05**	**81**
3	150	rt	1:1.0	30
4	200	rt	1:5.0	3
5	100	0	1:27	1
6	100	40	1:0.4	46
7	100	reflux	Not precipitated	-
8[d]	100	rt	Not precipitated	-

[a] (*S*)-**1a**, (*R*)-**1b**, and **2** were mixed in toluene at room temperature for 2 d. [b] Other macrocycles and oligomers. [c] Determined by ^{1}H NMR analysis. [d] AcOH (2% v/v) was added.

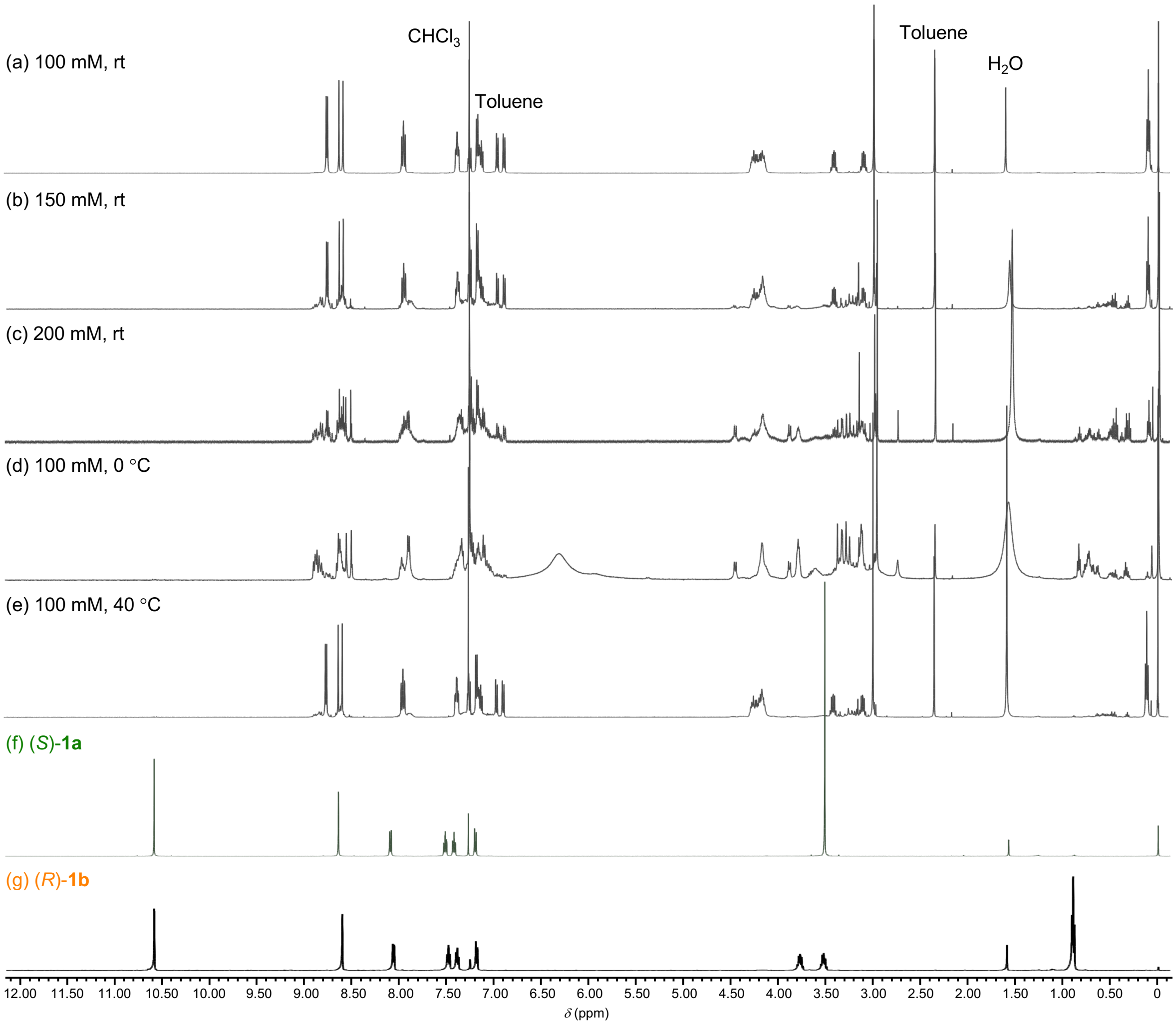

Figure S2. ^{1}H NMR spectra (500 MHz, in $CDCl_3$, rt) of macrocyclic and oligomeric imines precipitated from toluene in different conditions. (a) 100 mM, rt (Table S2, entry 2), (b) 150 mM, rt (Table S2, entry 3), (c) 200 mM, rt (Table S2, entry 4), (d) 100 mM, 0 °C (Table S2, entry 5), and (e) 100 mM, 40 °C (Table S2, entry 6). ^{1}H NMR spectra (500 MHz, in $CDCl_3$, rt) of (f) (*S*)-**1a** and (h) (*R*)-**1b**.

4. HRMS-ESI Analyses

The formation of macrocyclic imines (*S*,*R*,*S*,*R*)-**3** and (*S*,*R*)-**4** was confirmed by the HRMS-ESI analysis. Experimental and theoretical spectra are shown in Figures S3–S8.

<u>(*S*,*R*,*S*,*R*)-**3**</u>

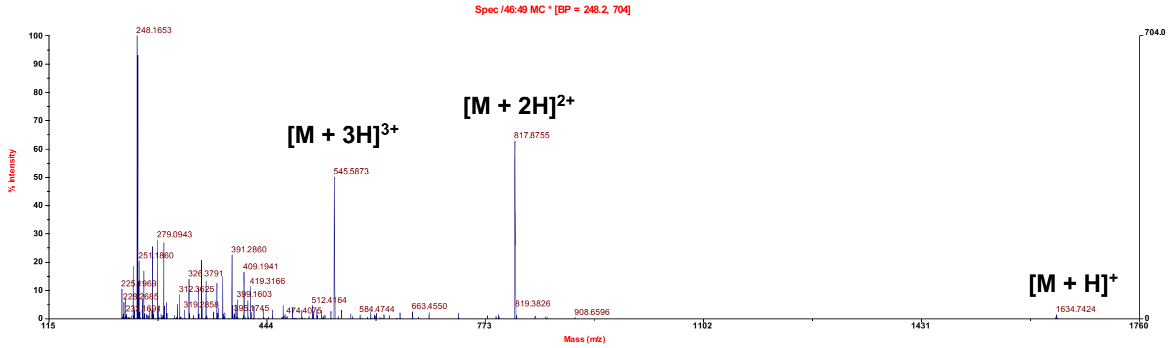

Figure S3. HRMS-ESI spectrum of (*S*,*R*,*S*,*R*)-**3**.

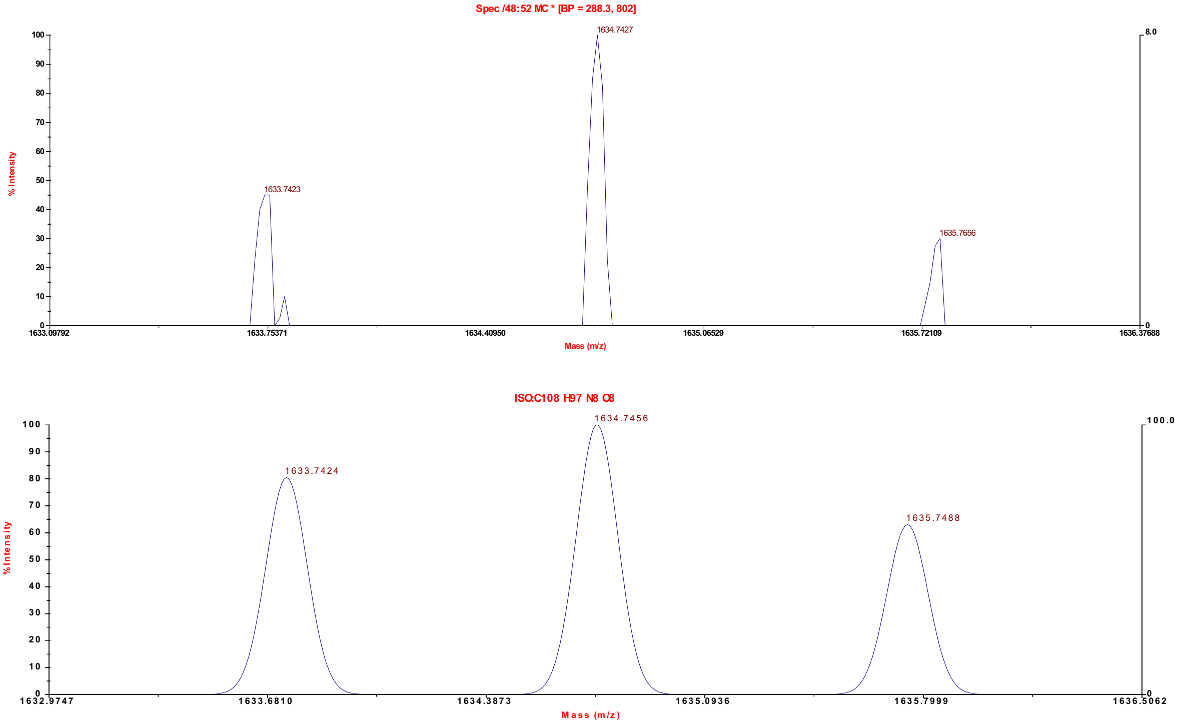

Figure S4. Observed (top) and theoretical (bottom) isotopic distribution peaks of the cation $[M + H]^+$ of (*S*,*R*,*S*,*R*)-**3**.

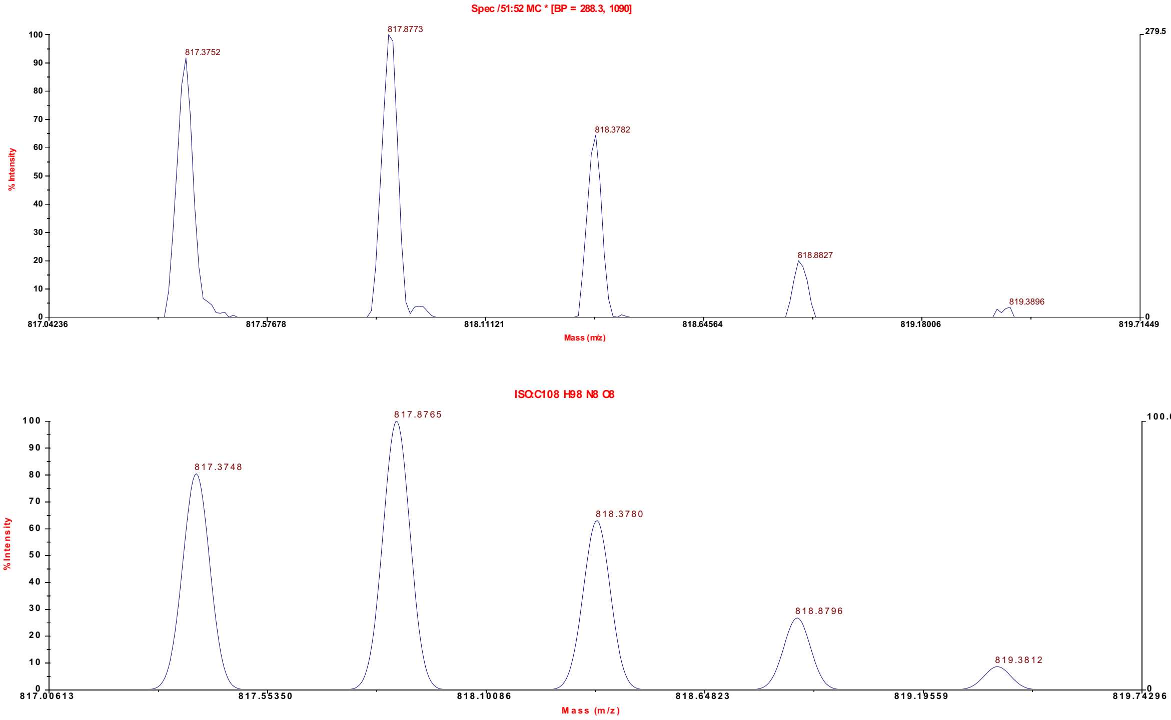

Figure S5. Observed (top) and theoretical (bottom) isotopic distribution peaks of the cation $[M + 2H]^{2+}$ of (*S*,*R*,*S*,*R*)-**3**.

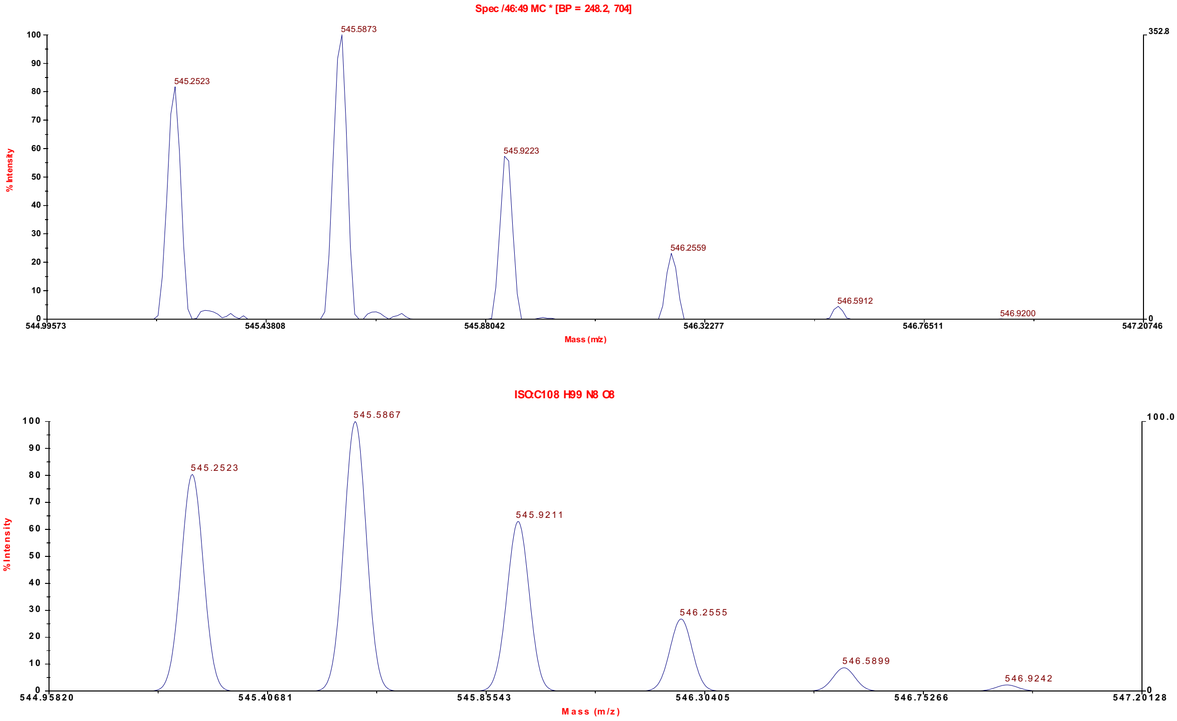

Figure S6. Observed (top) and theoretical (bottom) isotopic distribution peaks of the cation $[M + 3H]^{3+}$ of (*S*,*R*,*S*,*R*)-**3**.

(*S*,*R*)-**4**

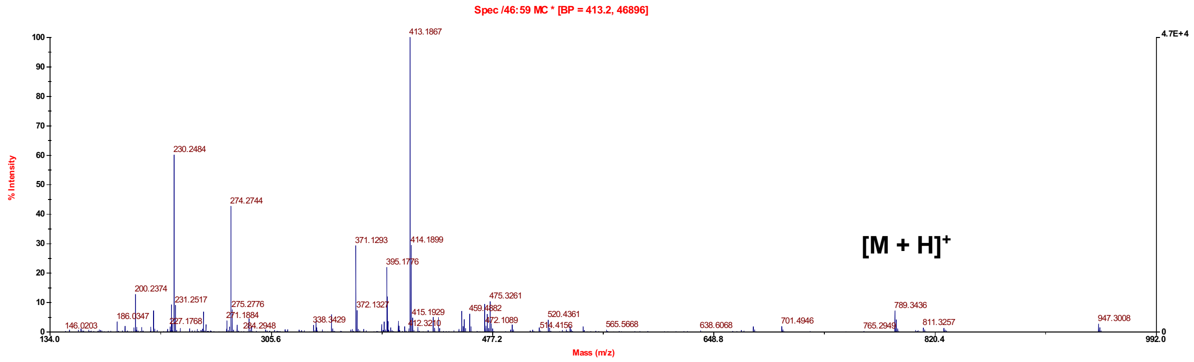

Figure S7. HRMS-ESI spectrum of (*S*,*R*)-**4**.

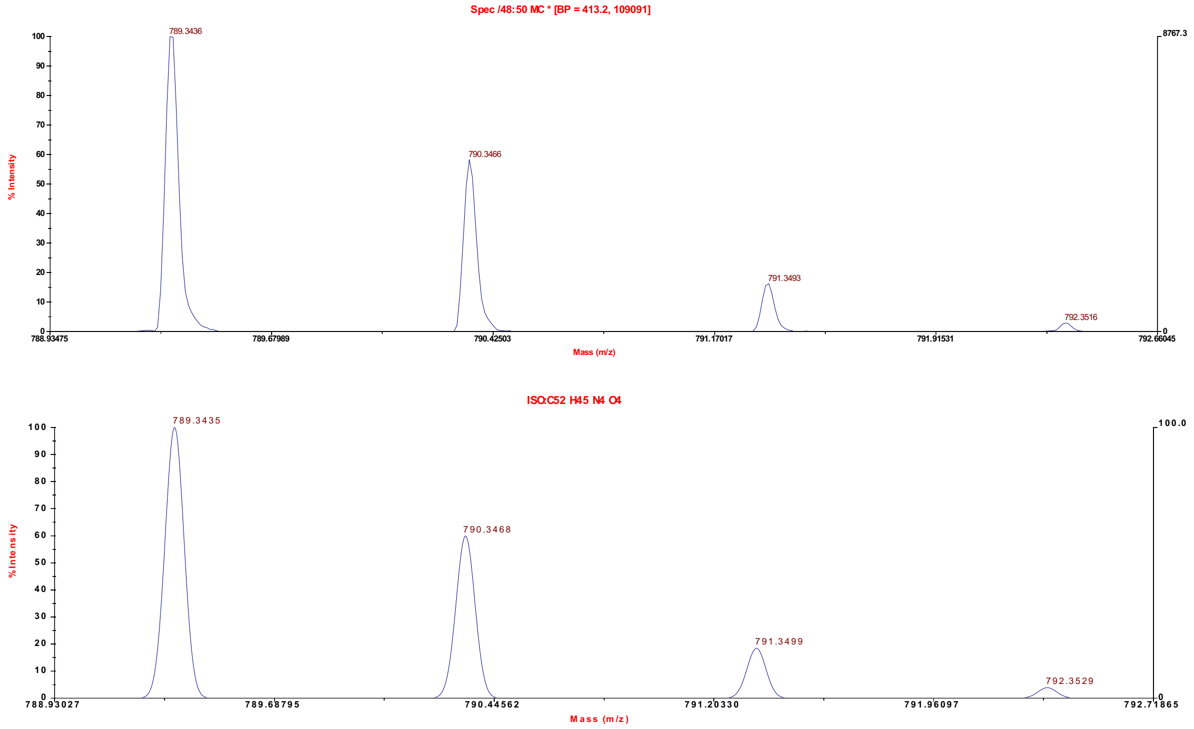

Figure S8. Observed (top) and theoretical (bottom) isotopic distribution peaks of the cation $[M + H]^+$ of (*S*,*R*)-**4**.

5. Temporal ^{1}H NMR Spectra of Supernatants and Precipitates

The temporal changes in the compounds present in both the precipitate and the solution during the formation of (*S*,*R*,*S*,*R*)-**3** from (*S*)-**1a**, (*R*)-**1b**, and **2** in toluene were monitored by ^{1}H NMR analyses. No precipitation was observed after 10 min, 30 min, 1 h, and 2 h. Therefore, the reaction mixture was concentrated under reduced pressure, and the residue was dissolved in $CDCl_3$ for ^{1}H NMR measurement (Figure S9). Precipitation was observed after stirring for 3 h, 6 h, 9 h, 12 h, 24 h. 36 h, 48 h, and 72 h. The precipitates were collected by filtration, and the filtrates were concentrated under reduced pressure. The collected precipitates and the concentrated residues were dissolved in $CDCl_3$, and their ^{1}H NMR spectra were recorded (Figure S9 and S10).

The precipitates were consistently composed of almost pure (*S*,*R*,*S*,*R*)-**3** throughout the duration from the initial precipitation at 3 h to 72 h (Figure S10). In contrast, no signal of (*S*,*R*,*S*,*R*)-**3** were detected in the concentrated residues; instead, broad signals suggesting the formation of oligomers were detected (Figure S9). When stirring times were 10 min and 30 min, signals of (*S*)-**1a** and (*R*)-**1b** were also observed (Figure S9a and S9b). These results suggest that (*S*,*R*,*S*,*R*)-**3** formed gradually in the solution and started to precipitate after around 3 h, with the composition of the precipitate remaining stable from its initial formation.

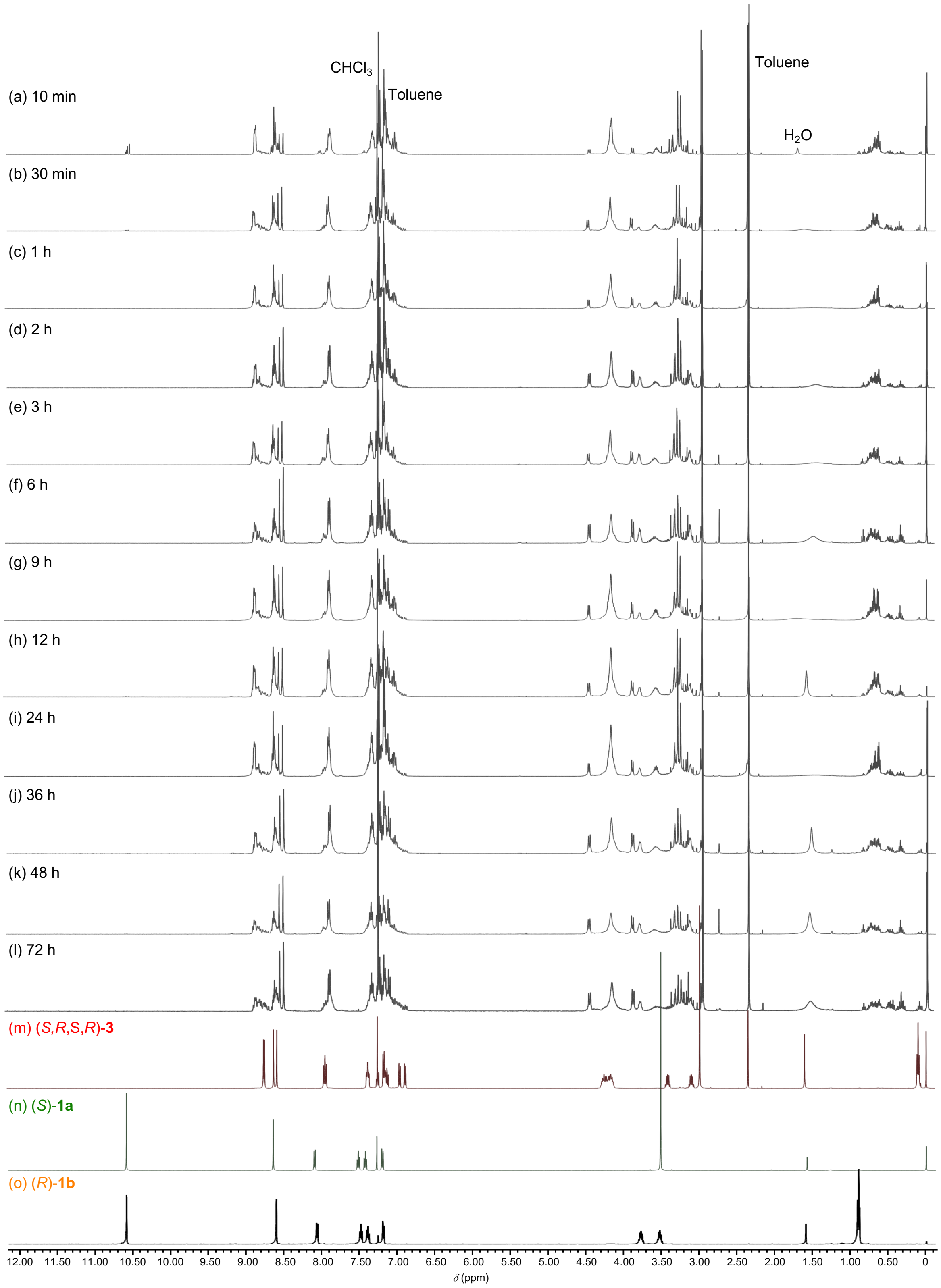

Figure S9. ^{1}H NMR spectra (500 MHz, in $CDCl_3$, rt) of the concentrated residues of the reaction mixture or filtrate obtained in the reaction of (*S*)-**1a**, (*R*)-**1b**, and **2** in toluene after (a) 10 min, (b) 30 min, (c) 1 h, (d) 2h, (e) 3 h, (f) 6 h, (g) 9 h, (h) 12 h, (i) 24 h, (j) 36 h, (k) 48 h, and (l) 72 h. ^{1}H NMR spectra (500 MHz, in $CDCl_3$, rt) of (m) (*S*,*R*,*S*,*R*)-**3**, (n) (*S*)-**1a**, and (o) (*R*)-**1b**.

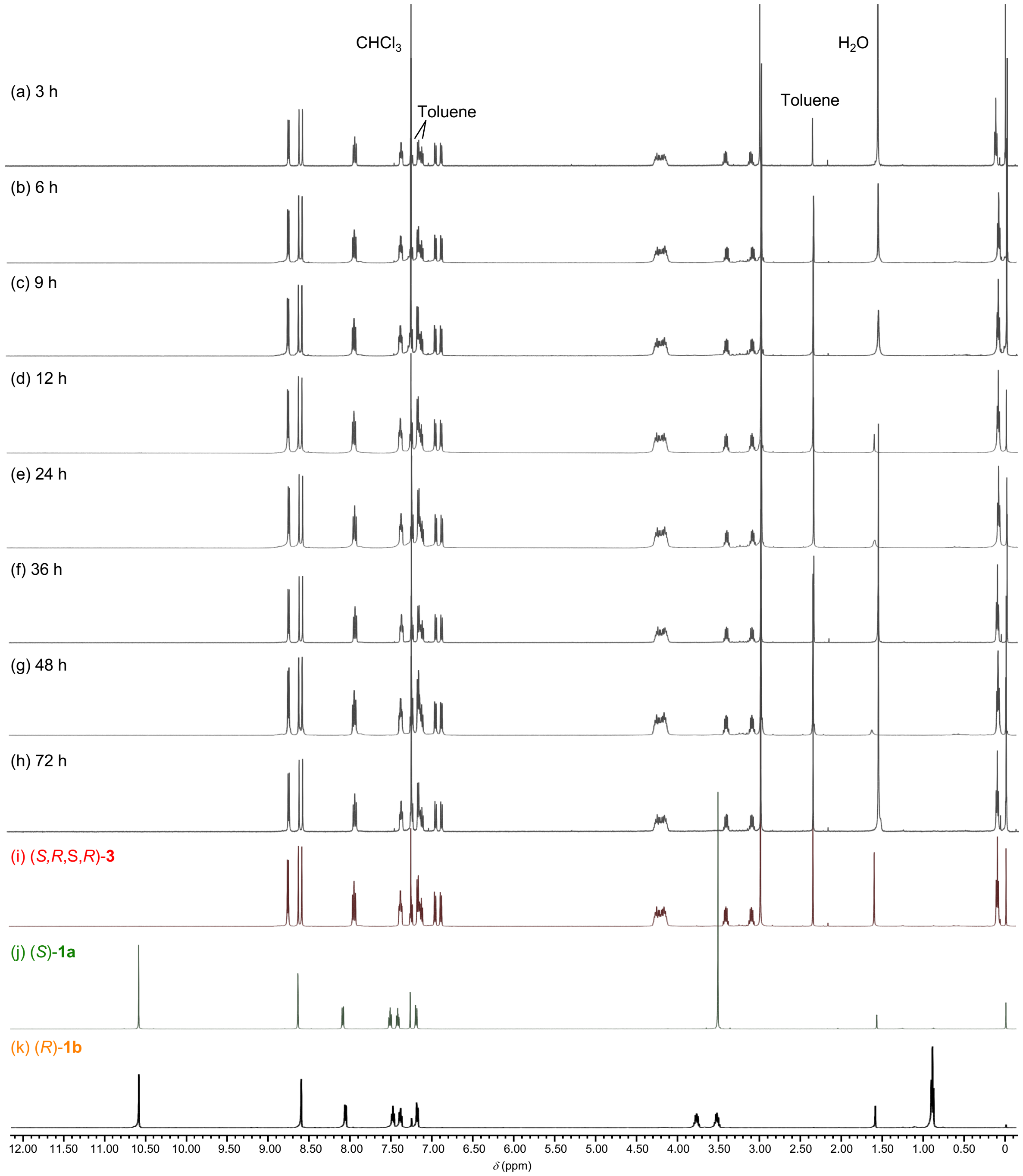

Figure S10. ^{1}H NMR spectra (500 MHz, in $CDCl_3$, rt) of the precipitates obtained in the reaction of (*S*)-**1a**, (*R*)-**1b**, and **2** in toluene after (a) 3 h, (b) 6 h, (c) 9 h, (d) 12 h, (e) 24 h, (f) 36 h, (g) 48 h, and (h) 72 h. ^{1}H NMR spectra (500 MHz, in $CDCl_3$, rt) of (i) (*S*,*R*,*S*,*R*)-**3**, (j) (*S*)-**1a**, and (k) (*R*)-**1b**.

6. Single-Crystal X-ray Diffraction Analyses

The crystalline samples were mounted on a glass fiber. All measurements were made on a Rigaku XtaLAB P200 diffractometer using multilayer mirror monochromated Cu–Kα radiation (λ = 1.54184 Å). The data were collected at a temperature of –150 ± 1 °C. The crystal-to-detector distance was 40.00 mm. Readout was performed in the 0.172 mm pixel mode. Data were collected and processed using CrysAlisPro (Rigaku Oxford Diffraction).[3] An empirical absorption correction was applied. The data were corrected for Lorentz and polarization effects. The structure of (*S*,*R*,*S*,*R*)-**3**·[solvent] was solved by direct methods (SIR92)[4] and expanded using Fourier techniques. The non-hydrogen atoms were refined anisotropically. Hydrogen atoms were refined using the riding model. All calculations of (*S*,*R*,*S*,*R*)-**3**·[solvent] were performed using the CrystalStructure[5] crystallographic software package except for refinement. Using Olex2,[6] the structure of (*S*,*R*,*S*,*R*)-**3**·2(1-butanol)·$2H_2O$·[solvent] was solved with the SHELXT[7] structure solution program using Intrinsic Phasing. The refinement was carried out with the SHELXL[8] refinement package using least squares minimization.

(*S*,*R*,*S*,*R*)-**3**·[solvent]

A single crystal of (*S*,*R*,*S*,*R*)-**3** suitable for X-ray crystallographic analysis was obtained by vapor diffusion of toluene into a chloroform solution of (*S*,*R*,*S*,*R*)-**3**. The electron density within the pores was eliminated using the SQUEEZE method.[9]

Crystal data (CCDC 2329858): $C_{108}H_{96}N_8O_8$, M = 1634.00, tetragonal, $a = b$ = 29.5388(3) Å, c = 11.85987(13) Å, V = 10348.22(19) Å^3, space group $I4_1$ (no. 80), Z = 4, D_c = 1.049 g cm^{-3}, $F(000)$ = 3456.00, T = 123(1) K, μ(Cu–Kα) = 5.259 cm^{-1}, 31150 reflections measured, 9454 independent (R_{int} = 0.0408). The final refinement converged to R_1 = 0.0363 for $I > 2.0\sigma(I)$, wR_2 = 0.1066 for all data.

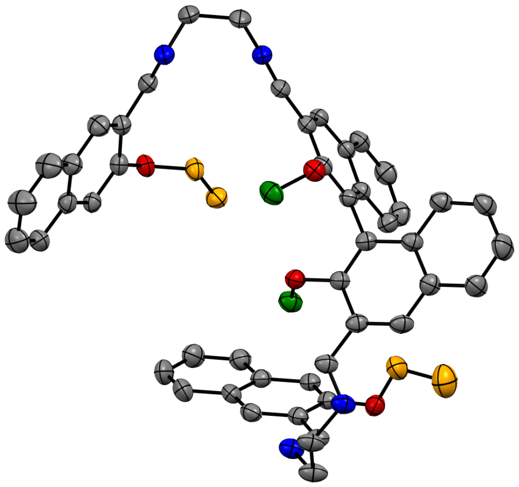

Figure S11. ORTEP structure (asymmetric unit) of (*S*,*R*,*S*,*R*)-**3** with atomic displacement parameters set at 50% probability [C = grey (Me groups = green, Et groups = orange), N = blue, O = red]. All hydrogen atoms are omitted for clarity.

Single crystal X-ray analysis confirms that the methyl and ethyl groups fill the internal space of the folded macrocyclic structure (Figure S12a).

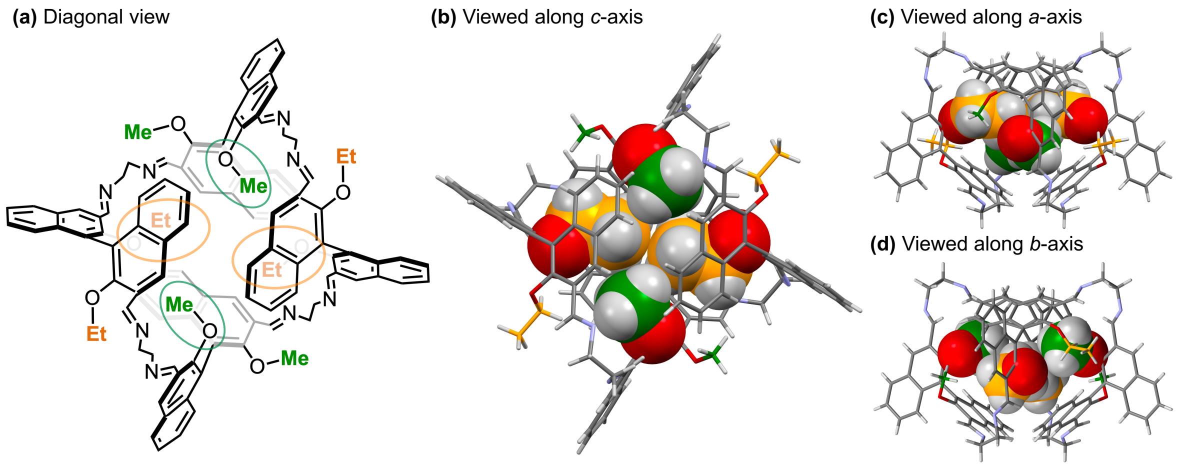

Figure S12. (a) Folded conformation of (*S*,*R*,*S*,*R*)-**3** in the crystalline state. (b–d) Single-crystal X-ray structures viewed along (b) *c*-axis, (c) *a*-axis, and (d) *b*-axis. Methoxy and ethoxy groups are depicted in CPK model [C = grey (Me groups = green, Et groups = orange), N = blue, O = red].

In the crystal structure of (*S*,*R*,*S*,*R*)-**3**, the naphthyl-CH=N groups of adjacent molecules stack to form layers extended along the *a*- and *b*- axes (Figure S13a–c). These layers are further stacked along the *c*-axis without any shift in the *ab* planes, resulting in the formation of the dual porous structure. The key to this stacking along the *c*-axis is the CH/π interaction, where the CH at the 6-position of the naphthalene ring interacts with the C=N of the molecule situated diagonally across along the *c*-axis (Figure S13d). Therefore, it can be stated that the formation of the dual porous crystalline structure is facilitated by π-stacking interactions between molecules in the *ab* plane direction and CH···C=N interactions between layers along the *c*-axis (Figure S13e).

The interaction between the CH of the naphthalene ring and the C of the imine moiety was also confirmed through Hirshfeld surface analysis by using the CrystalExplorer package V.21.3[10] (Figure S14). Examination of the contact contribution ratio between adjacent molecules revealed that the contribution of H···C interactions is 19.7%.

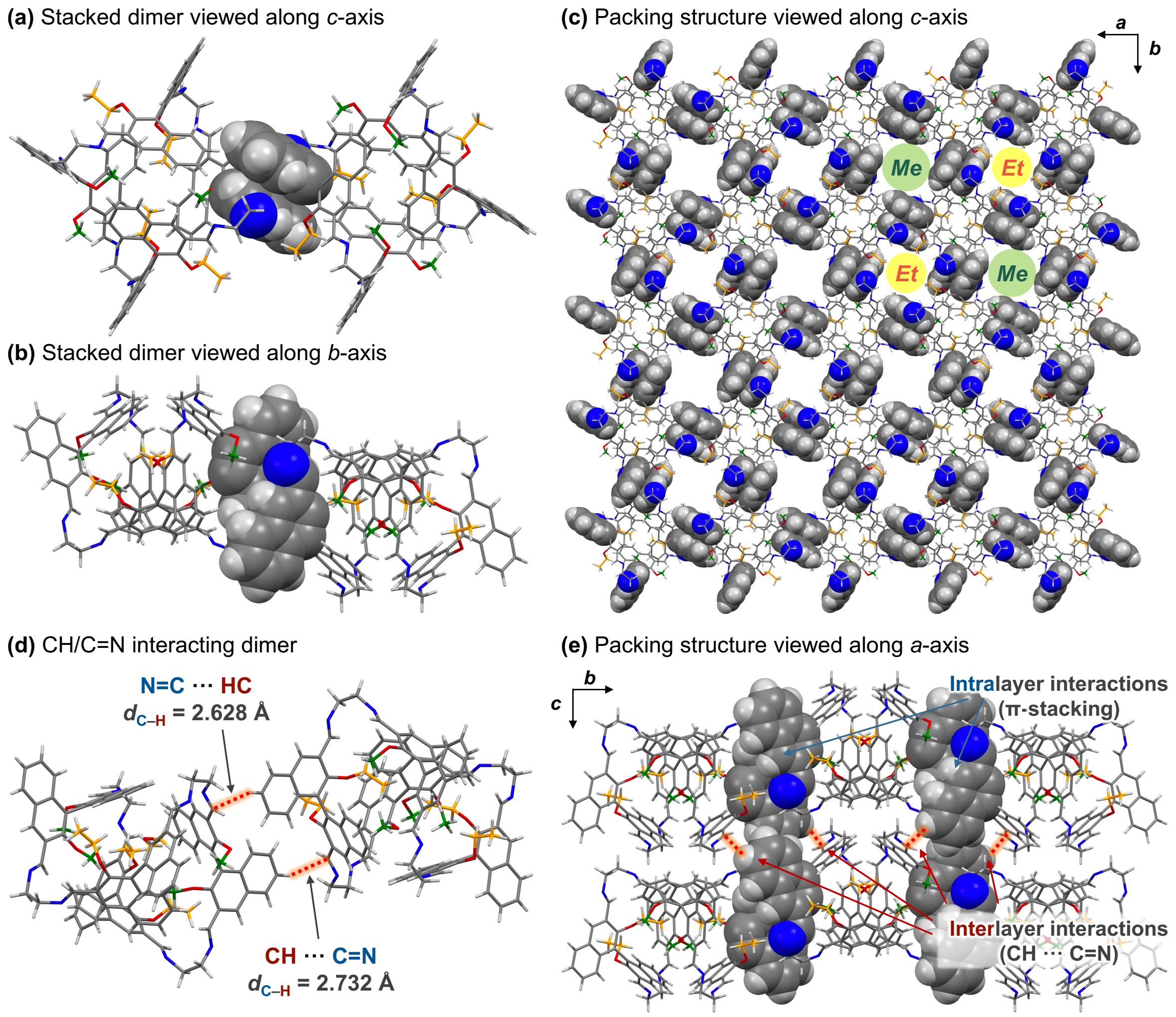

Figure S13. (a and b) Stacked dimer of (*S*,*R*,*S*,*R*)-**3** viewed along (a) *c*-axis (b) and *b*-axis. (c) Packing structure of (*S*,*R*,*S*,*R*)-**3** viewed along *c*-axis. (d) CH/C=N interacting dimer of (*S*,*R*,*S*,*R*)-**3**. (e) Packing structure of (*S*,*R*,*S*,*R*)-**3** viewed along *a*-axis. Naphthyl-CH=N groups are depicted in CPK model [C = grey (Me groups = green, Et groups = orange), N = blue, O = red].

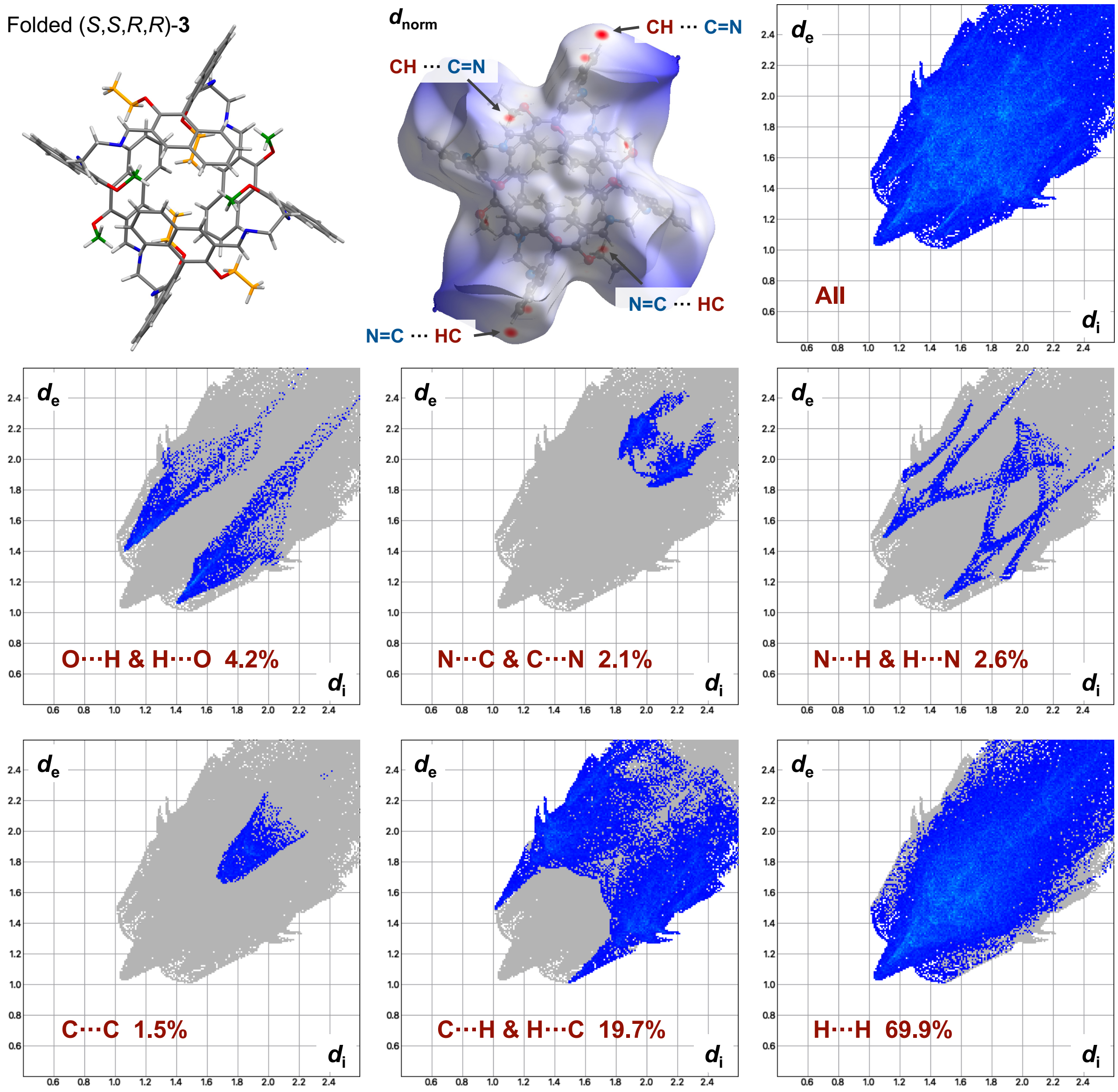

Figure S14. Folded molecular conformation, Hirshfeld surface mapped with normalized contact distance (d_{norm}), and two-dimensional fingerprint plots of various close intermolecular contacts of (*S*,*R*,*S*,*R*)-**3**.

7. Dynamic Imine Bonding Reactions of Racemic Precursors

7.1. Dynamic imine bonding between *rac*-**1a** and **2**

Under the same reaction conditions as the social self-sorting of (*S*,*R*,*S*,*R*)-**3**, (*S*)-**1a** and (*R*)-**1a** were stirred with **2** in toluene (100 mM) at room temperature for 2 d (Table S3, entry 1). The resulting precipitate was collected by filtration, and the ^{1}H NMR spectrum of the precipitate indicated the formation of a mixture of macrocyclic imines and oligomeric imines (*meso*-**4**/*rac*-**4**/oligomeric imines = 1.0 : <0.05 : 0.9). The NMR yield of the major macrocyclic imine, which was later identified as *meso*-**4** [= (*S*,*R*)-**4**], was 33%.

After screening of the cosolvent, almost pure *meso*-**4** was obtained in 78% yield as precipitates from toluene/CH_3CN = 2:1 mixed solvent system (entry 9: *meso*-**4**/*rac*-**4**/oligomeric imines = 1.0 : N.D. : N.D.). Based on the HRMS-ESI analysis (Figures S7 and S8), the macrocyclic imine **4** is composed of two molecules each of **1a** and **2**. There are two diastereomers for such macrocyclic imine: *meso*-**4** [= (*S*,*R*)-**4**], which is composed of socially self-sorted (*S*)-**1a** and (*R*)-**1a**, and *rac*-**4** [= (*S*,*S*)-**4** and (*R*,*R*)-**4**], which is composed of narcissistically self-sorted either (*S*)-**1a** or (*R*)-**1a**. When Et_2O, cyclohexane, or $CHCl_3$ was used as the cosolvent (entries 2–4), the formation of another diastereomer of macrocyclic imine **4** was confirmed in each case by HRMS-ESI analysis. The ^{1}H NMR spectrum of this diastereomer was in good agreement with that obtained from the reaction of (*S*)-**1a** and **2**, which should afford (*S*,*S*)-**4** (Figure S15). Accordingly, the macrocyclic imine formed in the self-sorting of *rac*-**1a** and **2** in toluene or toluene/CH_3CN was identified as (*S*,*R*)-**4**.

Table S3. Screening of the reaction conditions in the self-sorting of (*S*)-**1a** and (*R*)-**1a** into (*S*,*R*)-**4**.[a]

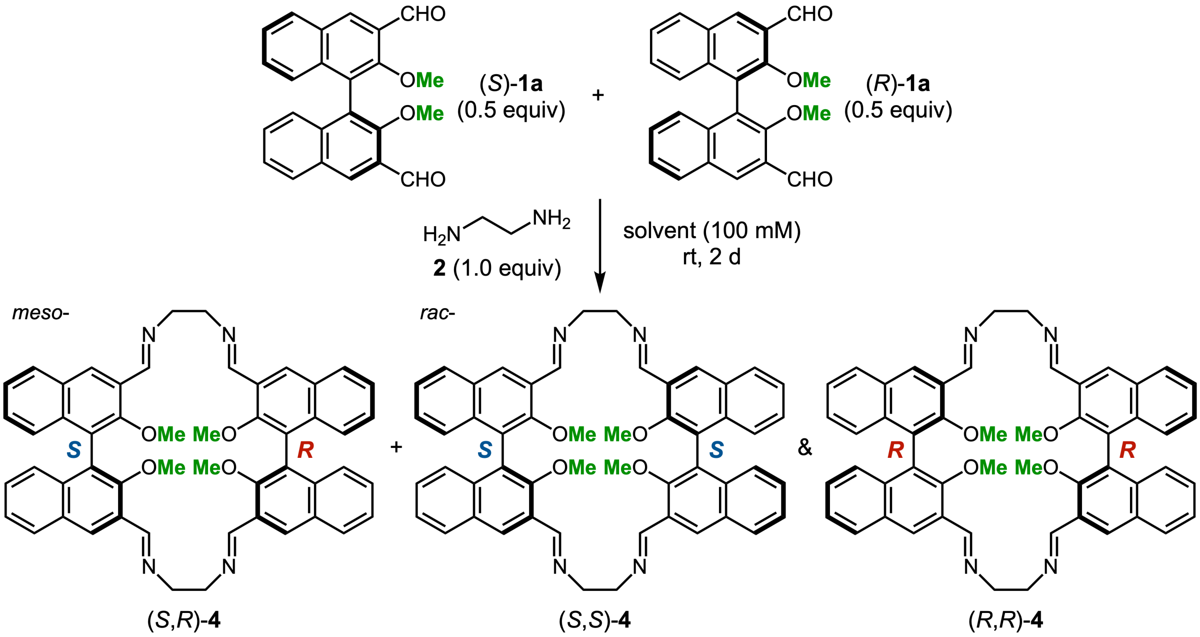

Entry	Solvent	*meso*-**4**/*rac*-**4**/others[b,c]	Yield of *meso*-**4** (%)[c]
1	Toluene	1.0 : <0.05 : 0.9	33
2	Toluene/Et_2O = 2:1	0.3 : 1.0 : 3.2	4
3	Toluene/Cyclohexane = 2:1	0.5 : 1.0 : 4.0	2
4	Toluene/$CHCl_3$ = 2:1	0.4 : 1.0 : 1.7	6
5	Toluene/EtOAc = 2:1	1.0 : 0.08 : 1.4	30
6	Toluene/THF= 2:1	1.0 : 0.05 :1.1	29
7	Toluene/EtOH = 2:1	1.0 : 0.1 : 0.7	44
8	Toluene/MeOH = 2:1	1.0 : <0.05 : 0.1	71
**9**	**Toluene/MeCN = 2:1**	**1.0 : N.D. : N.D.**	**78**

[a] (*S*)-**1a**, (*R*)-**1a**, and **2** were mixed in solvent (100 mM) at room temperature for 2 d. [b] Other macrocycles and oligomers. [c] Determined by ^{1}H NMR analysis.

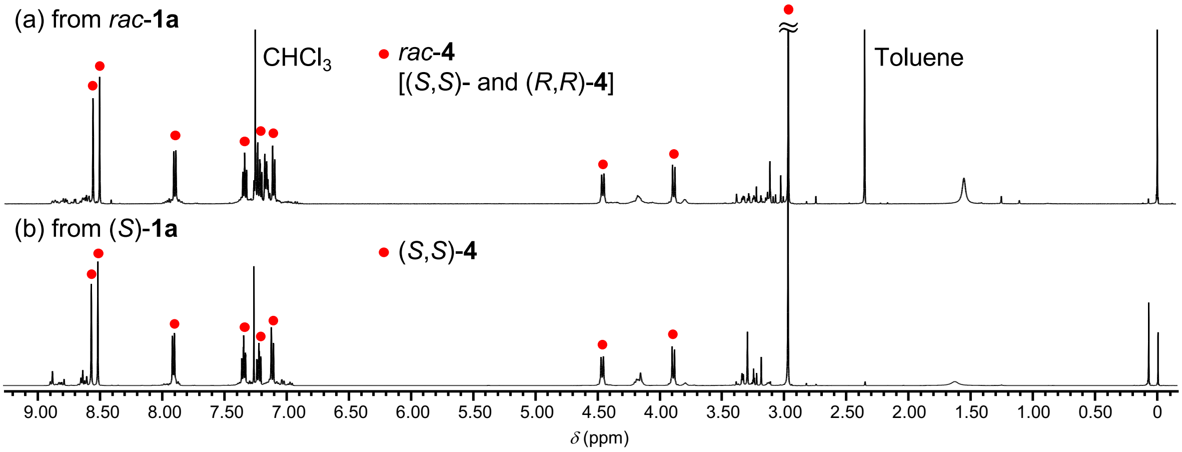

Figure S15. ^{1}H NMR spectra (500 MHz, in $CDCl_3$, rt) of the precipitates obtained in the rection of **2** with (a) *rac*-**1a** or (b) (*S*)-**1a**.

<u>7.2. Dynamic imine bonding between *rac*-**1b** and **2**</u>

Under the same reaction conditions as the social self-sorting of (*S*,*R*,*S*,*R*)-**3**, (*S*)-**1b** and (*R*)-**1b** were stirred with **2** in toluene (100 mM) at room temperature for 2 d. The resulting solution was concentrated under reduced pressure, and the ^{1}H NMR spectrum of the residue indicated the formation of an oligomeric imine mixture (Figure S16)。

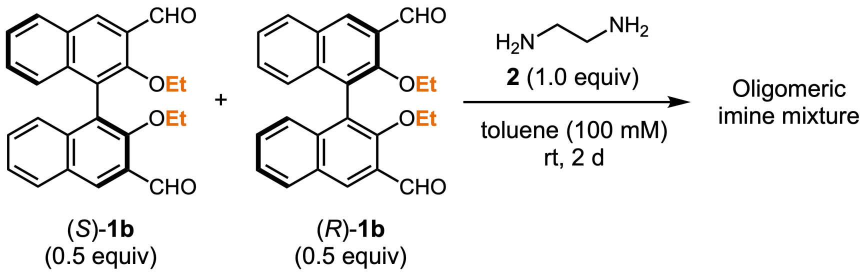

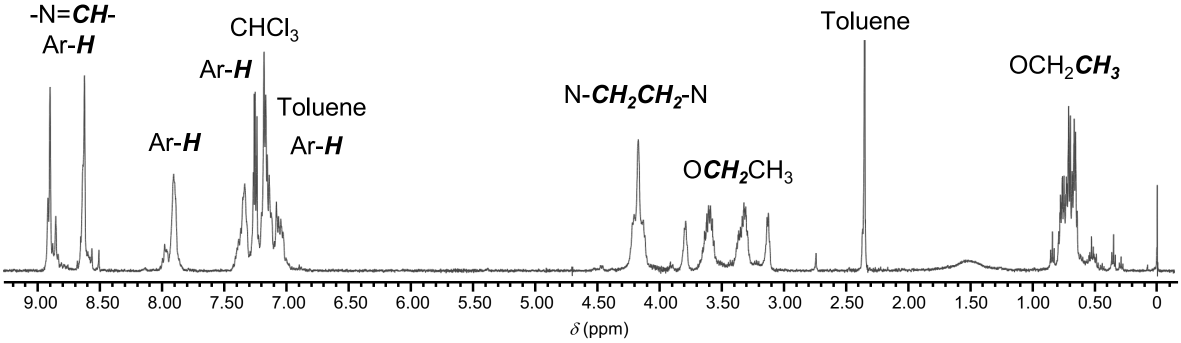

Figure S16. ^{1}H NMR spectrum (500 MHz, in $CDCl_3$, rt) of the oligomeric imine mixture obtained by the reaction of *rac*-**1b** and **2** in toluene (100 mM) at room temperature for 2 d.

7.3. Dynamic imine bonding using (*S*)-**1a**, (*S*)-**1b**, and **2**

Under the same reaction conditions as the social self-sorting of (*S*,*R*,*S*,*R*)-**3**, (*S*)-**1a** and (*S*)-**1b** were stirred with **2** in toluene (100 mM) at room temperature for 2 d. The resulting solution was concentrated under reduced pressure, and the ^{1}H NMR spectrum of the residue indicated the formation of an oligomeric imine mixture (Figure S17).

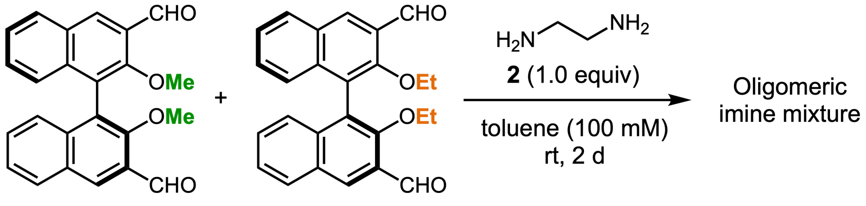

(*S*)-**1a**
(0.5 equiv)

(*S*)-**1b**
(0.5 equiv)

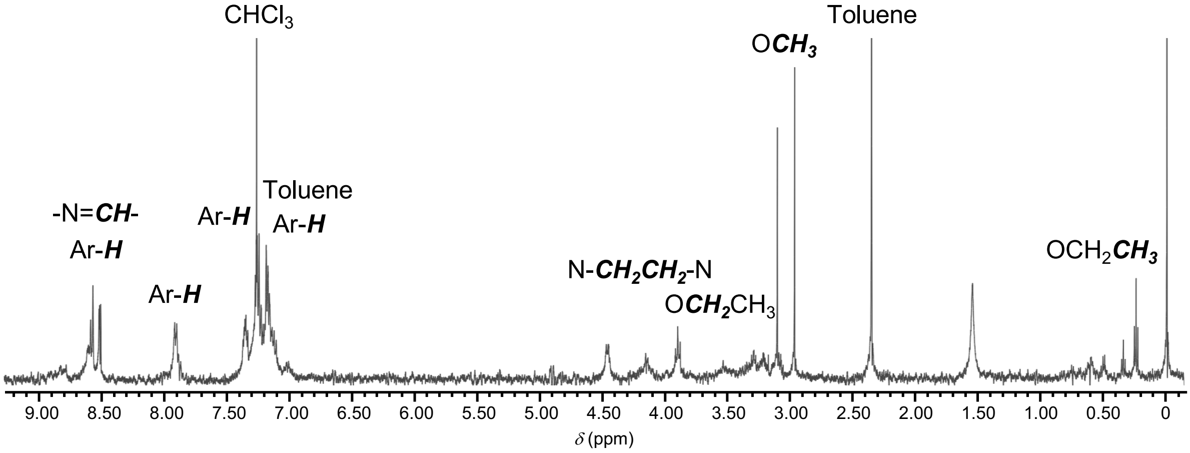

Figure S17. ^{1}H NMR spectrum (500 MHz, in $CDCl_3$, rt) of the oligomeric imine mixture obtained by the reaction of (*S*)-**1a**, (*S*)-**1b**, and **2** in toluene (100 mM) at room temperature for 2 d.

8. Nitrogen Adsorption Isotherm Measurement of (*S*,*R*,*S*,*R*)-3

8.1. ^{1}H NMR spectrum of heated (*S*,*R*,*S*,*R*)-**3**

The precipitated powder sample of (*S*,*R*,*S*,*R*)-**3**·toluene was heated at 100 °C for 1 h. The ^{1}H NMR spectrum of the heated (*S*,*R*,*S*,*R*)-**3** showed the complete removal of the included toluene molecules (Figure S18).

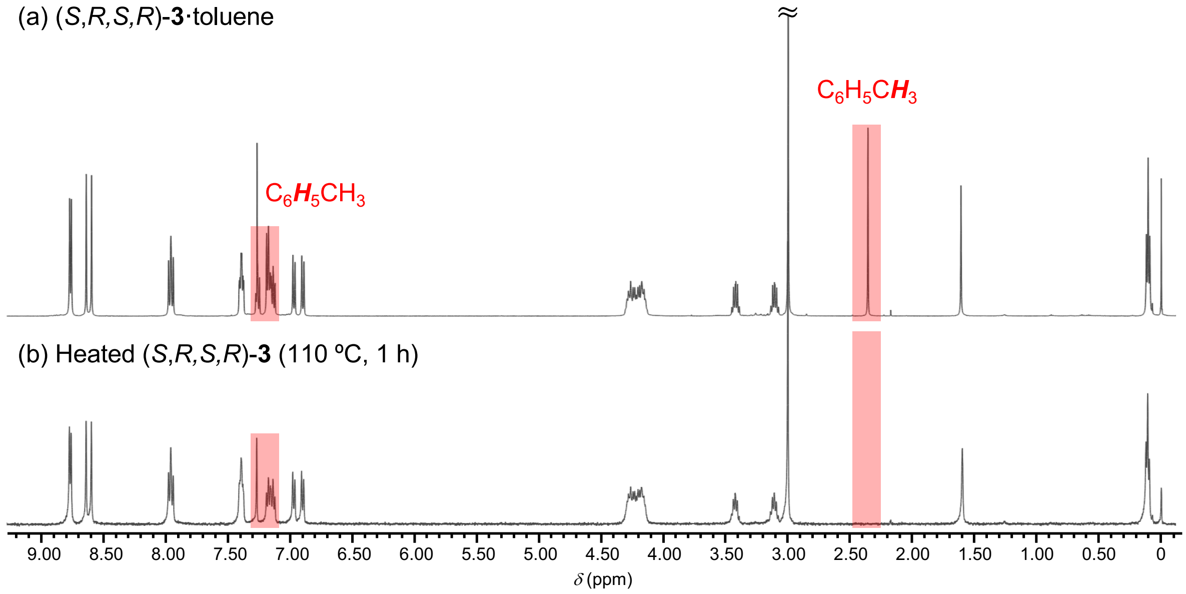

Figure S18. ^{1}H NMR spectra (500 MHz, in $CDCl_3$, rt) of (a) (*S*,*R*,*S*,*R*)-**3**·toluene and (b) heated (*S*,*R*,*S*,*R*)-**3**.

8.2. Nitrogen adsorption isotherm measurement

The two 1D micropores of crystalline (*S*,*R*,*S*,*R*)-**3** were characterized on the basis of multipoint N_2 adsorption isotherm (Figure S19a and S19c) acquired at 77.35 K using a gas sorption analyzer (BELSORP-MAX X, MicrotracBEL). The crystalline powder of (*S*,*R*,*S*,*R*)-**3**·toluene was heated *in vacuo* at 110 °C for 3 h prior to the measurement. The specific surface area was obtained from the adsorption isotherm using the Brunauer–Emmett–Teller (BET) equation (Figure S19b).[11,12] Data in the relative pressure range 0.0005–0.02 were used for the surface area evaluations.[12] The each micropore volume of (*S*,*R*,*S*,*R*)-**3** was calculated from the adsorption isotherm by the α_S-plot method (Figure S19d).[12,13] The micropore size distribution in crystalline (*S*,*R*,*S*,*R*)-**3** was calculated using Saito-Foley (SF) method (Figure 3c).[12,14]

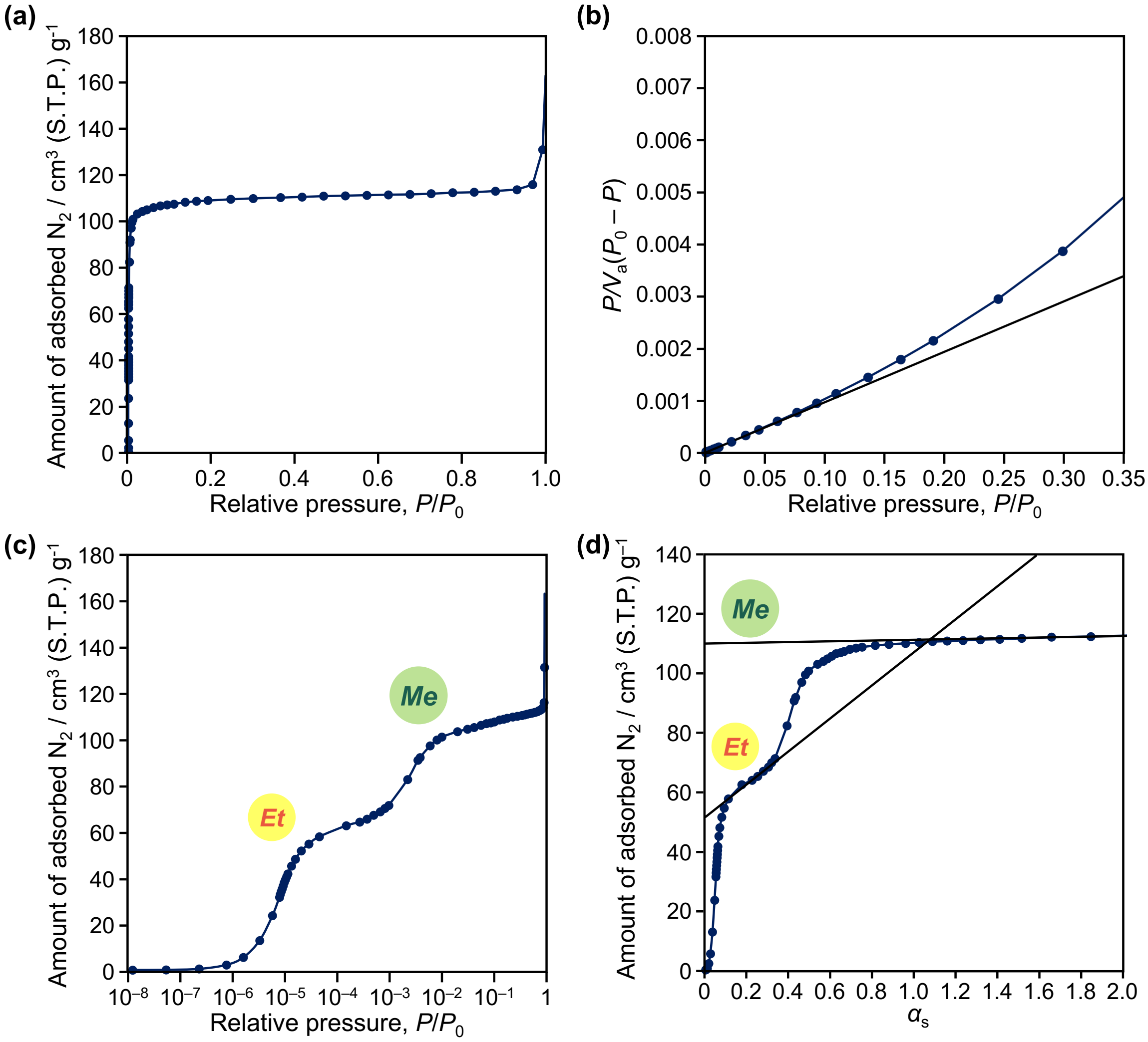

Figure S19. (a) Nitrogen adsorption isotherm (77 K) of (*S*,*R*,*S*,*R*)-**3**. (b) BET plot for (*S*,*R*,*S*,*R*)-**3** calculated from the nitrogen adsorption isotherm (77 K). (c) Nitrogen adsorption isotherm (77 K) of (*S*,*R*,*S*,*R*)-**3** in semi-logarithmic-scale plot. (d) α_s-plot derived from the nitrogen adsorption isotherm (77 K) of (*S*,*R*,*S*,*R*)-**3**.

9. Adsorption of Alcohol Vapors

For the alcohol vapor adsorption experiment, a crystalline sample of (*S*,*R*,*S*,*R*)-**3**·toluene and guest alcohol were independently introduced into individual microtubes, which were then placed into a single sealed sample bottle. The samples were subsequently allowed to equilibrate at room temperature for 1 week, and then the alcohol-exposed (*S*,*R*,*S*,*R*)-**3** was dissolved in $CDCl_3$ for measuring ^1H NMR spectra. Initially, crystalline powders of (*S*,*R*,*S*,*R*)-**3**·toluene were employed for the adsorption experiment of various alcohols. When several alcohols shown in Figure S20 were used, ^1H NMR analyses indicated the presence of alcohol molecules in molar ratios ranging from 1:2 to 1:8 relative to (*S*,*R*,*S*,*R*)-**3** (Figure S21).

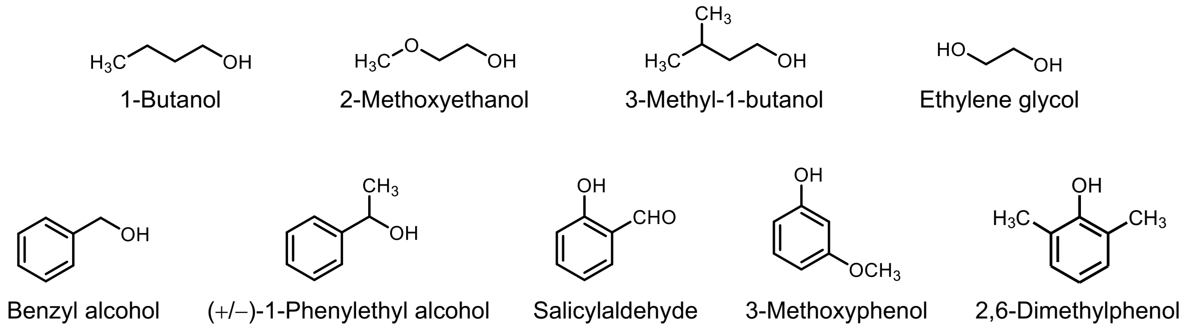

Figure S20. Guest molecules examined for vapor adsorption within the 1D micropores of single crystals of (*S*,*R*,*S*,*R*)-**3**·toluene.

The alcohol vapor adsorption experiment was then carried out using single crystals of (*S*,*R*,*S*,*R*)-**3**·toluene and alcohols shown in Figure S20. The structure of included alcohol molecules in the pores could be determined by X-ray diffraction analysis only when 1-butanol served as the guest alcohol. 1-Butanol molecules, along with H_2O molecules, were included in the larger pores of crystalline (*S*,*R*,*S*,*R*)-**3**. The molecular structure of 1-butanol in the smaller pores could not be determined due to disorder, and the electron density within the smaller pores was subsequently eliminated using the SQUEEZE method.[9]

Crystal data of (*S*,*R*,*S*,*R*)-**3**·2(1-butanol)·$2H_2O$·[solvent] (CCDC 2329859): $C_{116}H_{120}N_8O_{12}$, M = 1818.19, tetragonal, $a = b = 29.8339(2)$ Å, $c = 11.82350(10)$ Å, $V = 10523.64(17)$ Å^3, space group $I4_1$ (no. 80), $Z = 4$, $D_c = 1.148$ g cm^{-3}, $F(000) = 3872.00$, $T = 123(2)$ K, μ(Cu-Kα) = 5.91 cm^{-1}, 35696 reflections measured, 10593 independent ($R_{int} = 0.0252$). The final refinement converged to $R_1 = 0.0345$ for $I > 2.0\sigma(I)$, $wR_2 = 0.0998$ for all data.

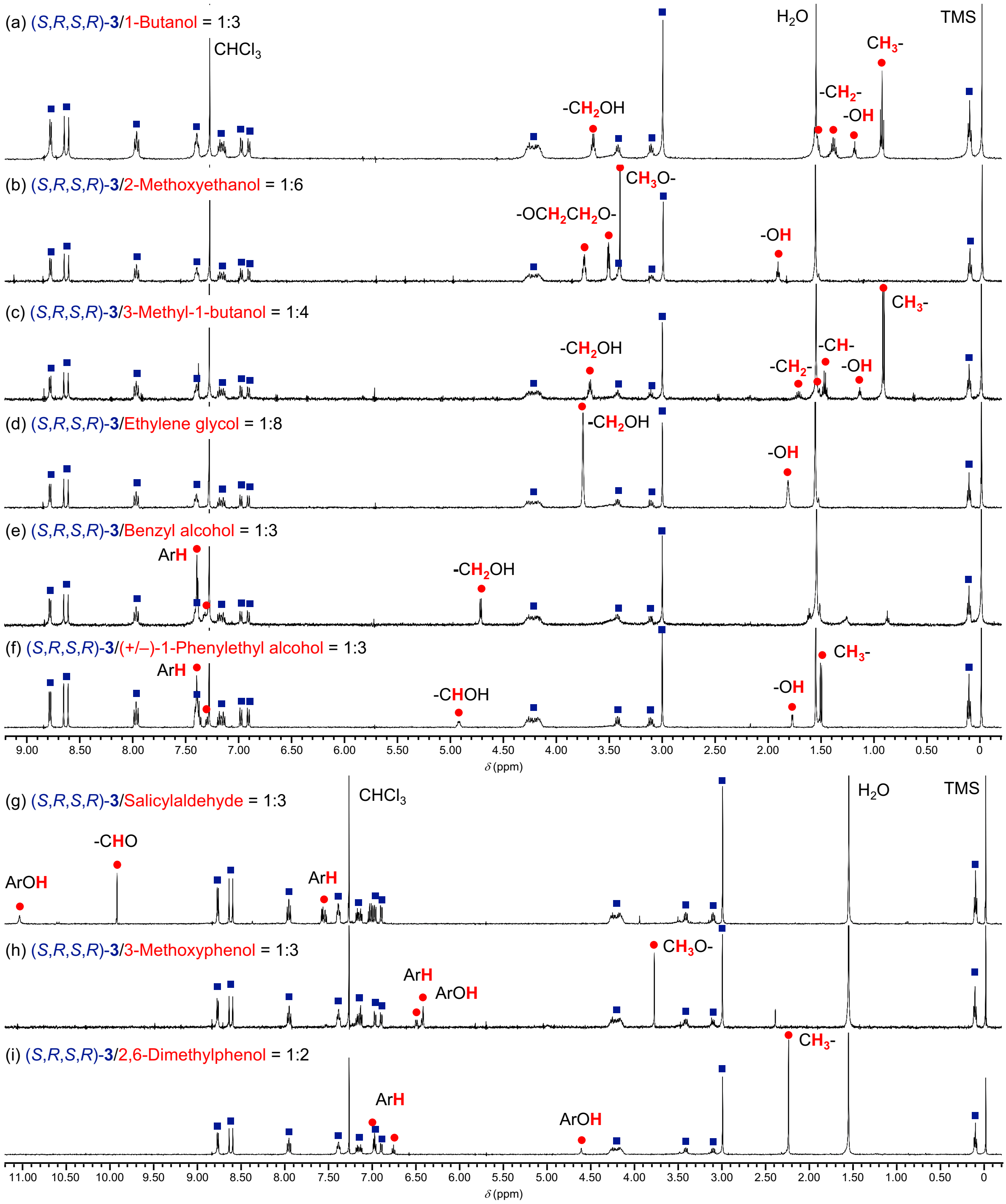

Figure S21. ^{1}H NMR spectra (500 MHz, in $CDCl_3$, rt) of (*S*,*R*,*S*,*R*)-**3** after exposure to (a–f) alcohol or (g–i) phenol vapors. The signals of (*S*,*R*,*S*,*R*)-**3** and guest molecules are indicated as blue squares and red circles, respectively.

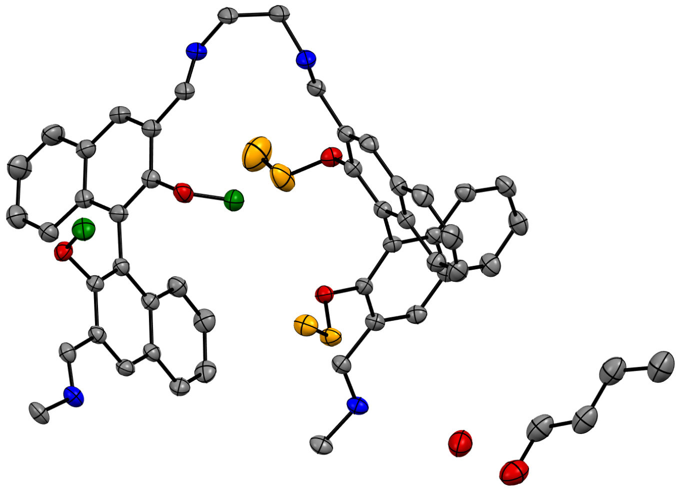

Figure S22. ORTEP structure (asymmetric unit) of (*S*,*R*,*S*,*R*)-**3**·2(1-butanol)·2H_2O·[solvent] with atomic displacement parameters set at 50% probability [C = grey (Me groups = green, Et groups = orange), N = blue, O = red]. All hydrogen atoms are omitted for clarity.

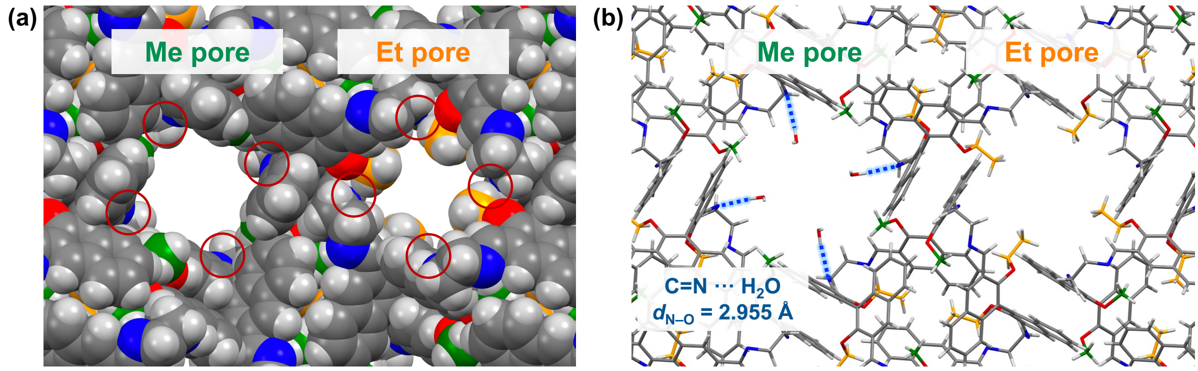

Figure S23. (a) Single-crystal structure of the two types of pores of (*S*,*R*,*S*,*R*)-**3** depicted in CPL model. Nitrogen atoms are indicated by red circles. (b) Single-crystal structure for the two types of pores in (*S*,*R*,*S*,*R*)-**3** depicted in capped sticks model. Hydrogen bonds between the imine groups of (*S*,*R*,*S*,*R*)-**3** and H_2O molecules are indicated. 1-Butanol molecules are omitted for clarity.

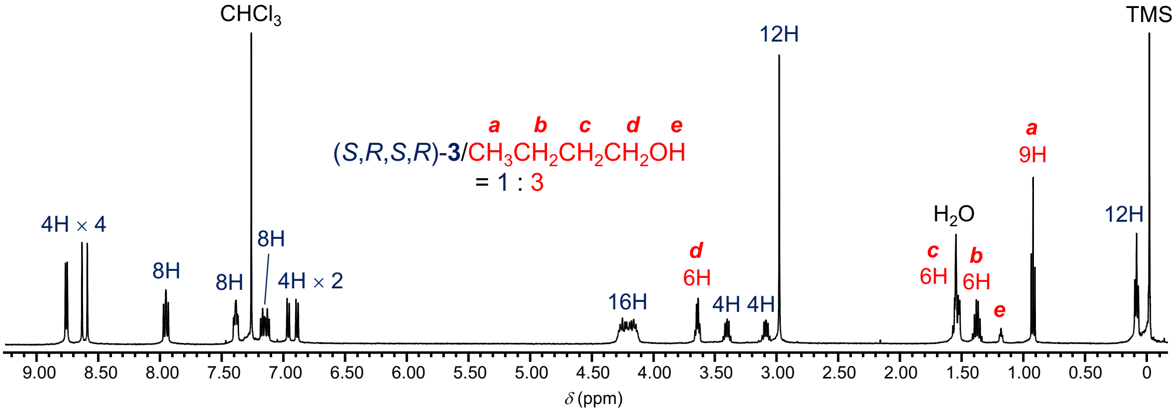

Figure S24. ^1H NMR spectrum (500 MHz, in $CDCl_3$, rt) of (*S*,*R*,*S*,*R*)-**3**·1-butanol·H_2O.

10. References

1) Lim, J. Y. C.; Marques, I.; Ferreira, L.; Félix, V.; Beer, P. D. Enhancing the enantioselective recognition and sensing of chiral anions by halogen bonding. *Chem. Commun.* **2016**, *52*, 5527.

2) Li, X.; Li, Q.; Wang, Y.; Quan, Y.; Chen, D.; Cheng, Y. Strong Aggregation-Induced CPL Response Promoted by Chiral Emissive Nematic Liquid Crystals (N*-LCs). *Chem. Eur. J.* **2018**, *24*, 12607.

3) *CrysAlisPro: Data Collection and Processing Software*; Rigaku Corporation: Tokyo, Japan, 2015.

4) Altomare, A.; Cascarano, G.; Giacovazzo, C.; Guagliardi, A. Completion and Refinement of Crystal Structures with *SIR*92. *J. Appl. Crystallogr.* **1993**, *26*, 343.

5) *CrystalStructure 4.2.5: Crystal Structure Analysis Package*; Rigaku Corporation: Tokyo, Japan, 2000–2017.

6) Dolomanov, O. V.; Bourhis, L. J.; Gildea, R. J.; Howard, J. A. K.; Puschmann, H. *OLEX2*: A Complete Structure Solution, Refinement and Analysis Program. *J. Appl. Crystallogr.* **2009**, *42*, 339.

7) Sheldrick, G. M. SHELXT - Integrated Space-Group and Crystal-Structure Determination. *Acta Crystallogr. A, Found. Adv.* **2015**, *71*, 3.

8) Sheldrick, G. M. Crystal Structure Refinement with *SHELXL*. *Acta Crystallogr., Sect. C: Struct. Chem.* **2015**, *C71*, 3.

9) van der Sluis, P.; Spek, A. L. BYPASS: an effective method for the refinement of crystal structures containing disordered solvent regions. *Acta Crystallogr., Sect. A* **1990**, *46*, 194.

10) Spackman, P. R.; Turner, M. J.; McKinnon, J. J.; Wolff, S. K.; Grimwood, D. J.; Jayatilaka, D.; Spackman, M. A. CrystalExplorer: a program for Hirshfeld surface analysis, visualization and qu-antitative analysis of molecular crystals. *J. Appl. Cryst.* **2021**, *54*, 1006.

11) Brunauer, S.; Emmett, P. H.; Teller, E. Adsorption of Gases in Multimolecular Layers. *J. Am. Chem. Soc.* **1938**, *60*, 309.

12) Thommes, M.; Kaneko, K; Neimark, A. V.; Olivier, J. P.; Rodriguez-Reinoso, F.; Rouquerol, J.; Sing, K. S. W. Physisorption of gases, with special reference to the evaluation of surface area and pore size distribution (IUPAC Technical Report). *Pure Appl. Chem.* **2015**, *87*, 1051.

13) Rouquerol, F.; Rouquerol, J.; Sing, K. S. W. Assessment of Surface Area. In *Adsorption by Powders & Porous Solids;* Pergamon Press, 1999; pp. 165–189.

14) Saito, A.; Foley, H. C. Curvature and parametric sensitivity in models for adsorption in micropores. *AIChE J.* **1991**, *37*, 429.

^{1}H NMR spectrum of (*S*,*R*,*S*,*R*)-**3**·2toluene (500 MHz, $CDCl_3$, rt)

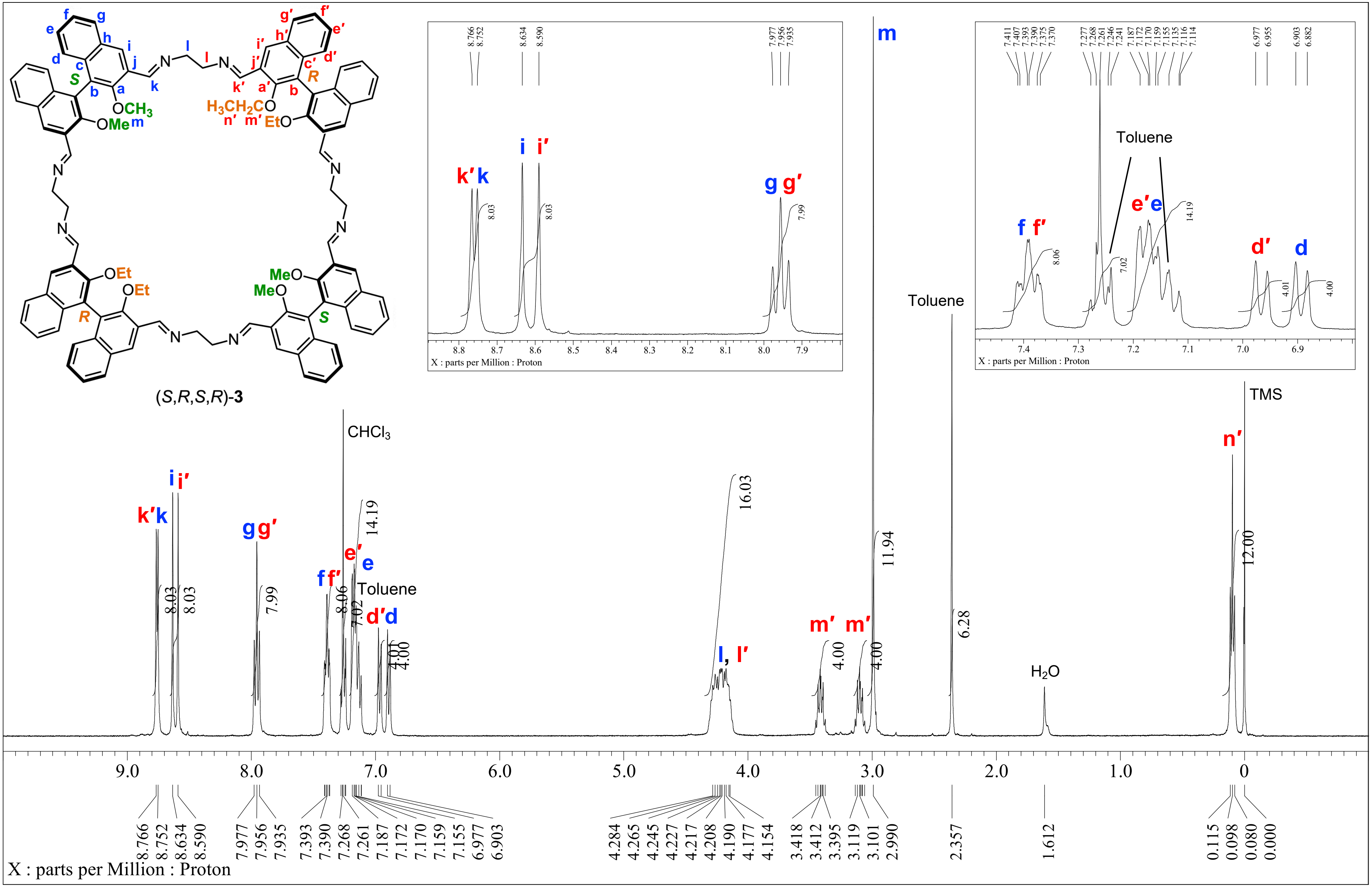

^{13}C NMR spectrum of (*S*,*R*,*S*,*R*)-**3**·2toluene (126 MHz, $CDCl_3$, –20 °C)

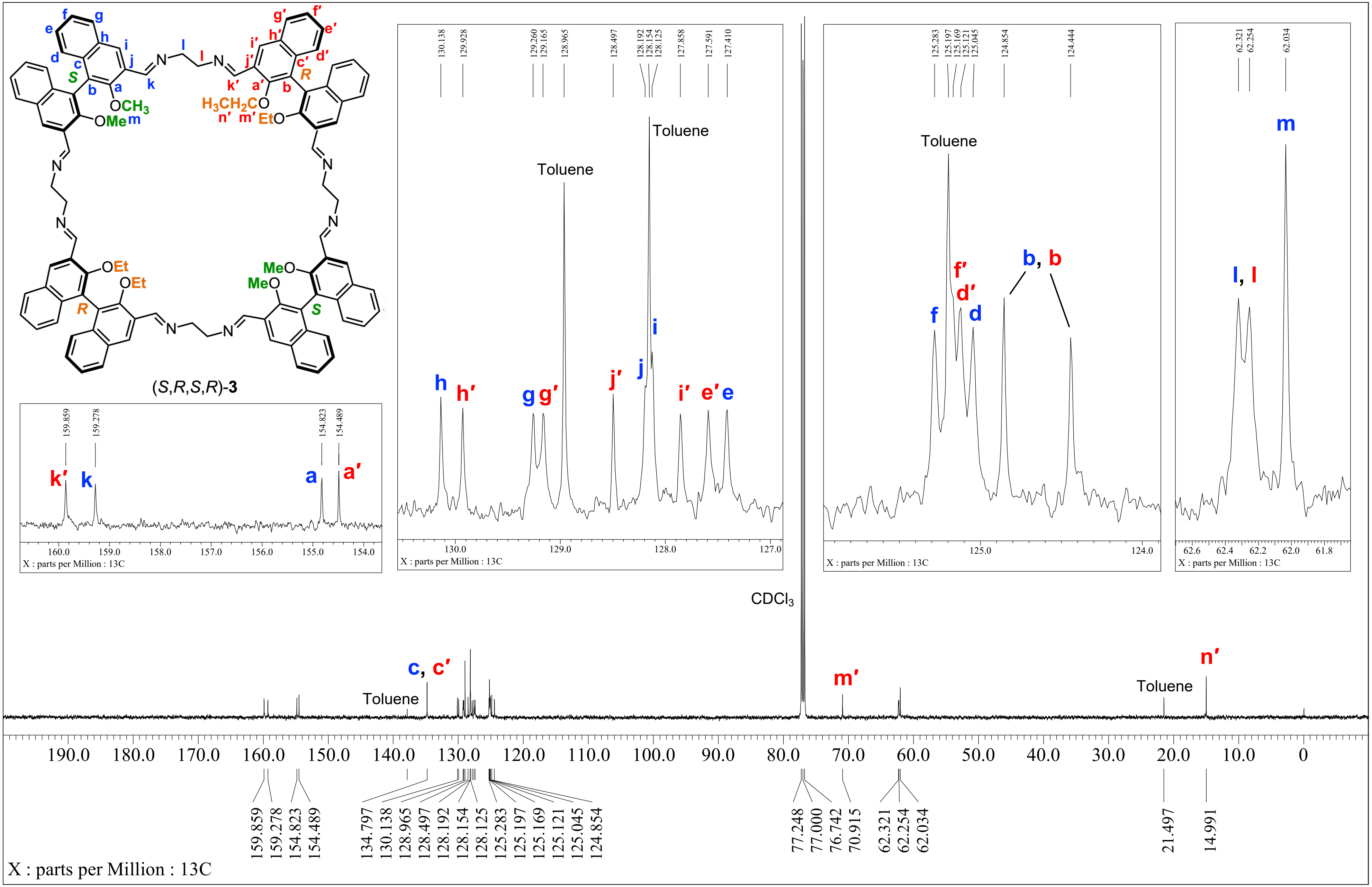

Partial ^1H-^1H COSY NMR spectrum of (*S*,*R*,*S*,*R*)-**3**·2toluene (500 MHz, $CDCl_3$, –20 °C)

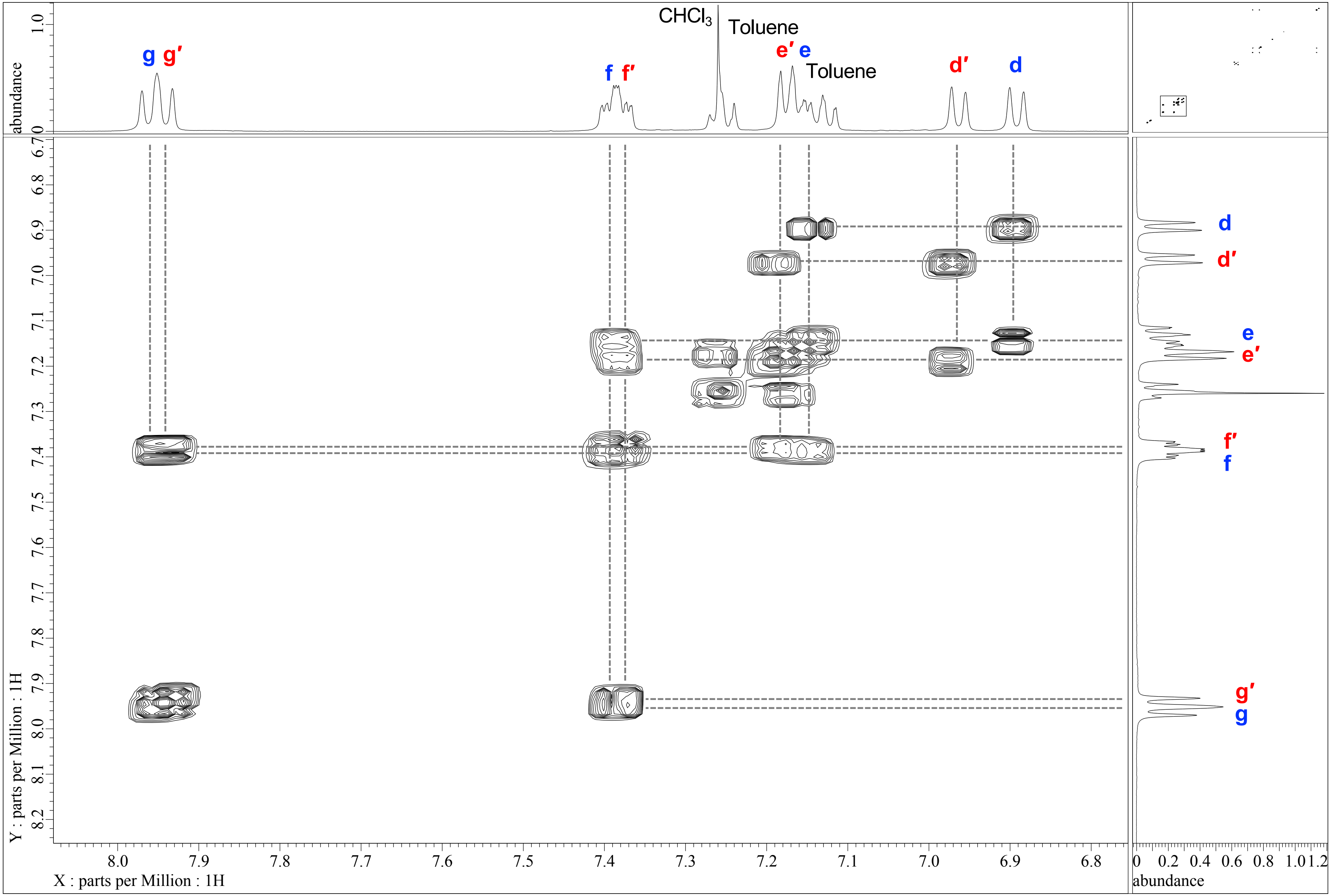

Partial HSQC NMR spectrum of (*S*,*R*,*S*,*R*)-**3**·2toluene (500 MHz, $CDCl_3$, –20 °C)

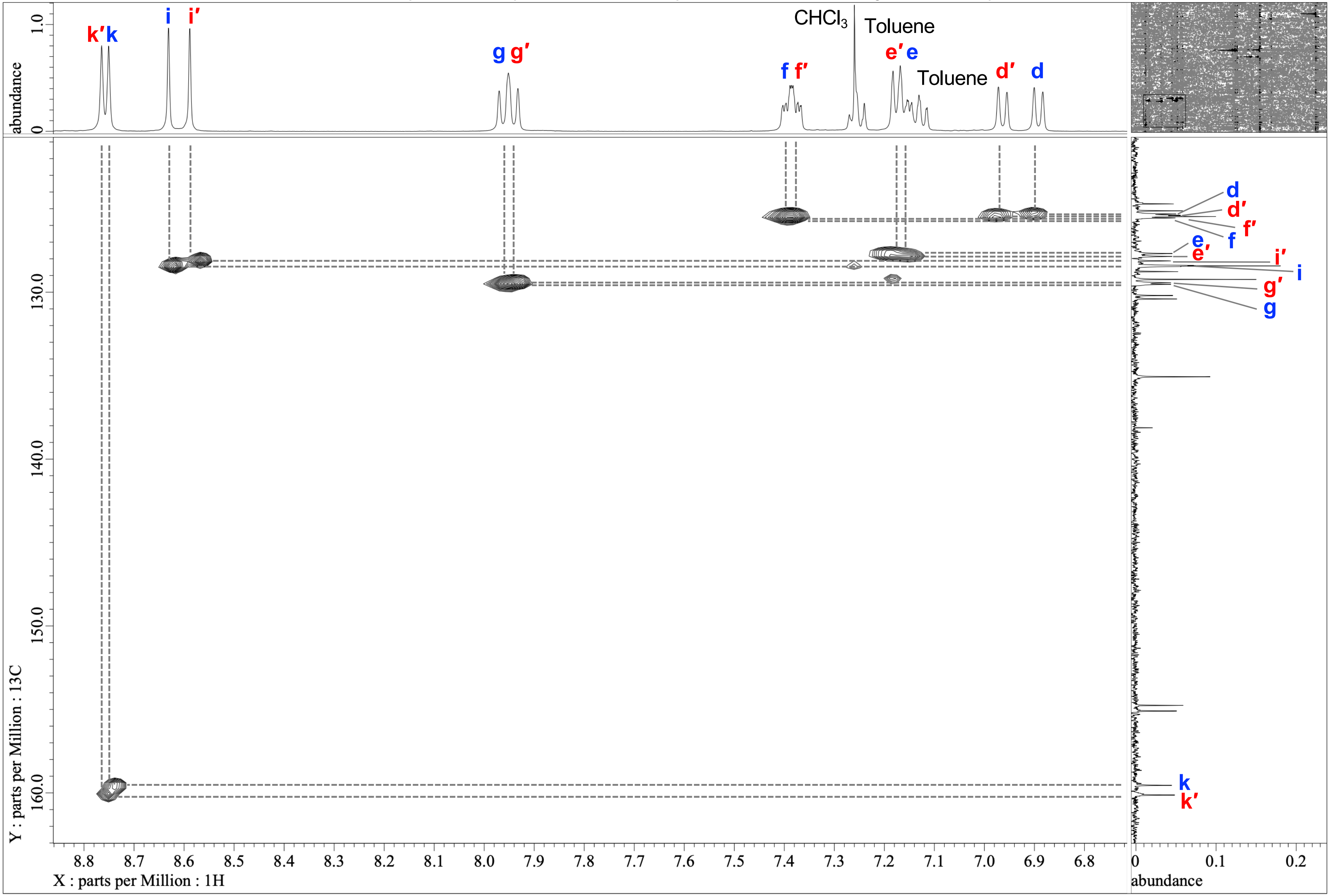

Partial HMBC NMR spectrum of (*S*,*R*,*S*,*R*)-**3**·2toluene (500 MHz, $CDCl_3$, –20 °C)

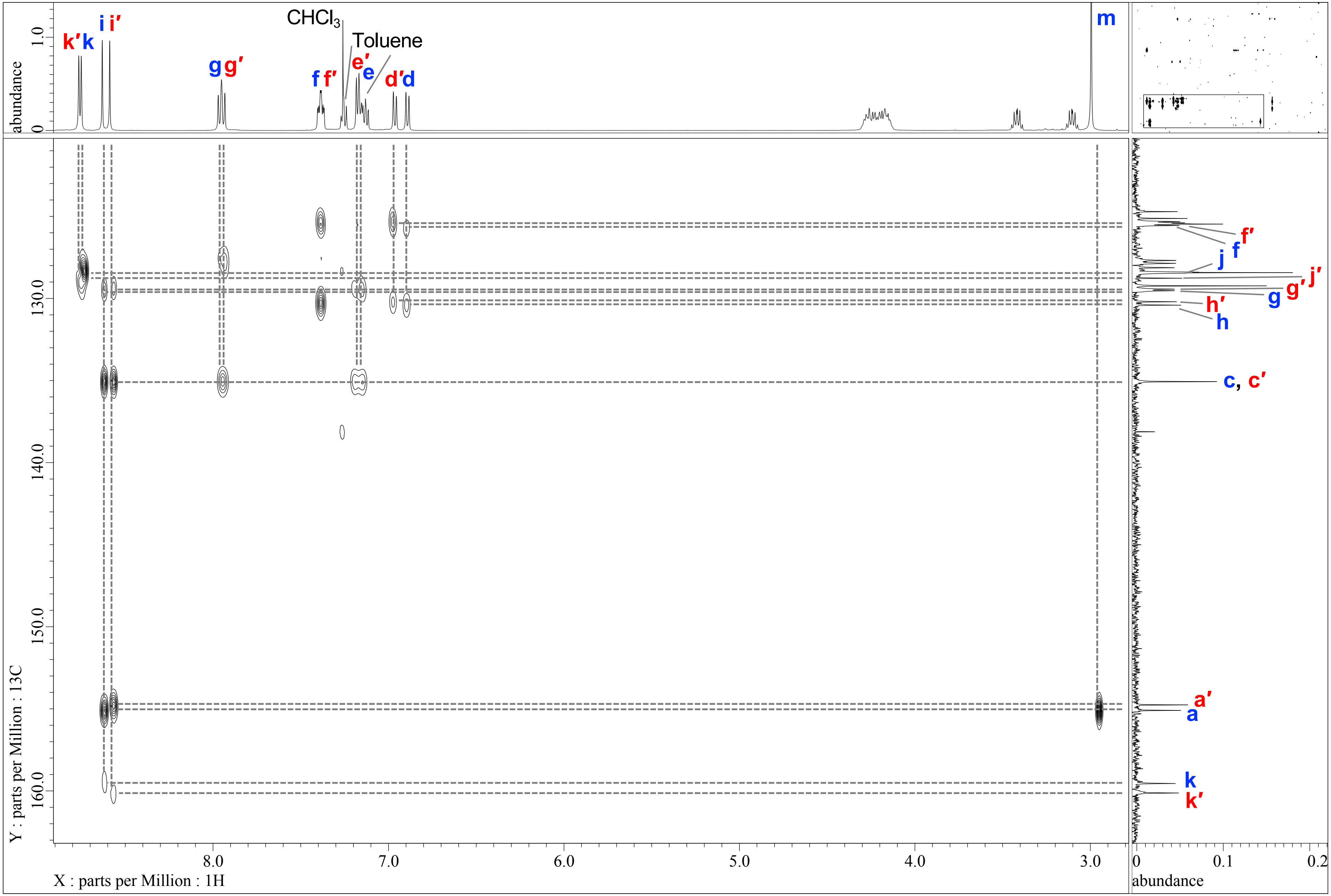

^1H NMR spectrum of (*S*,*R*)-**4** (500 MHz, $CDCl_3$, rt)

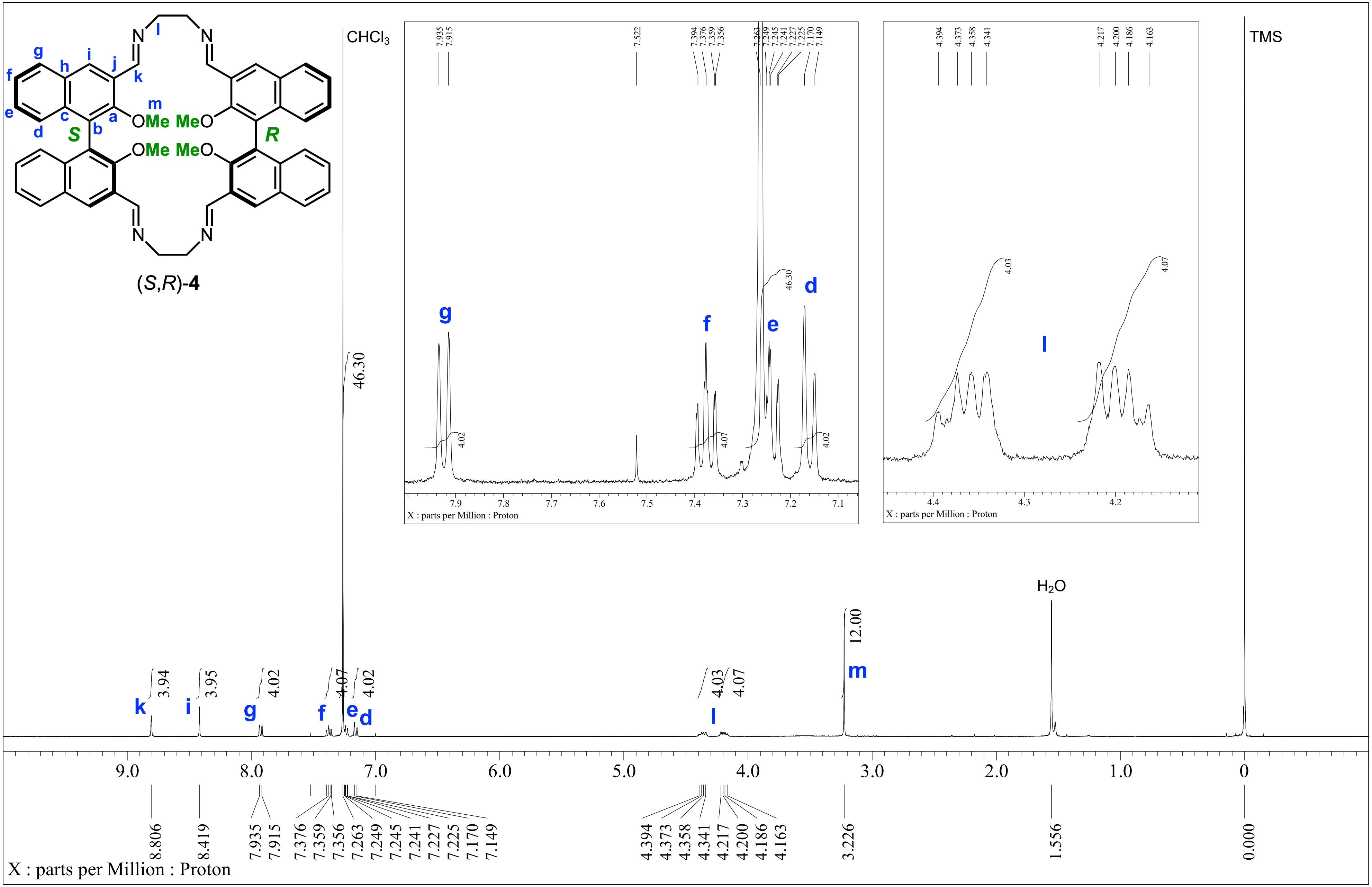

^{13}C NMR spectrum of (*S*,*R*)-**4** (126 MHz, $CDCl_3$, rt)

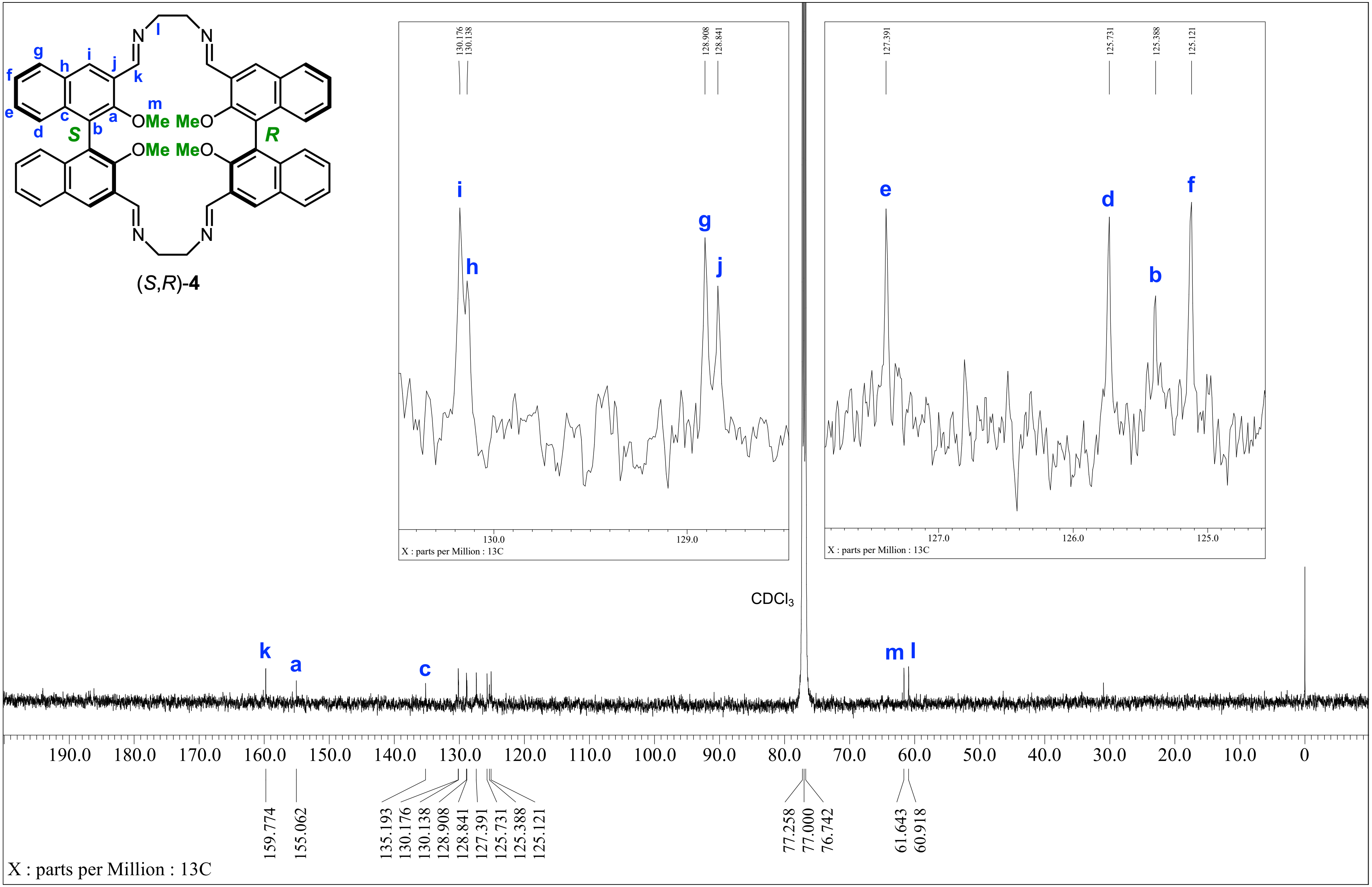

Partial ^{1}H-^{1}H COSY NMR spectrum of (*S*,*R*)-**4** (500 MHz, $CDCl_3$, rt)

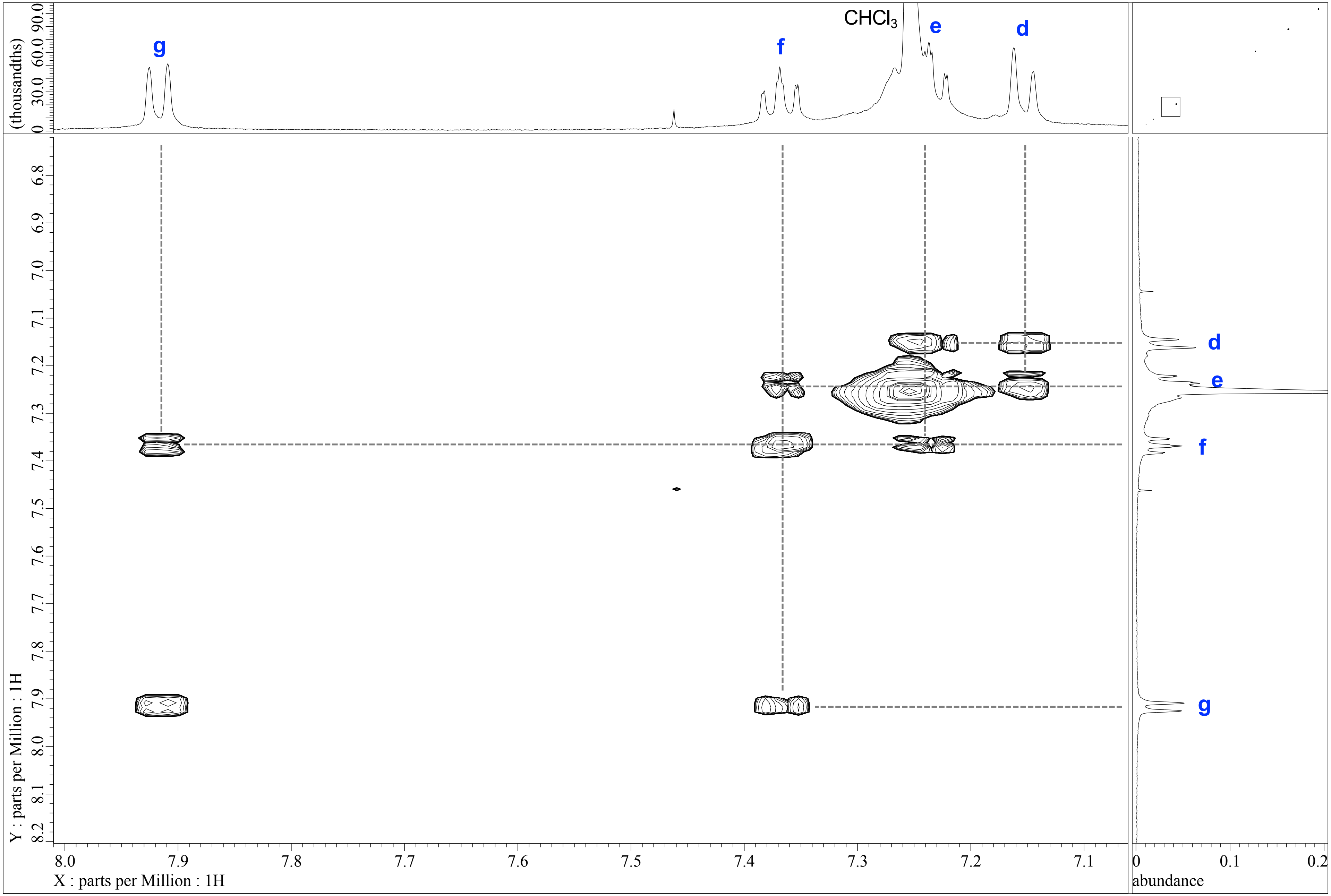

Partial HSQC NMR spectrum of (*S*,*R*)-**4** (500 MHz, $CDCl_3$, rt)

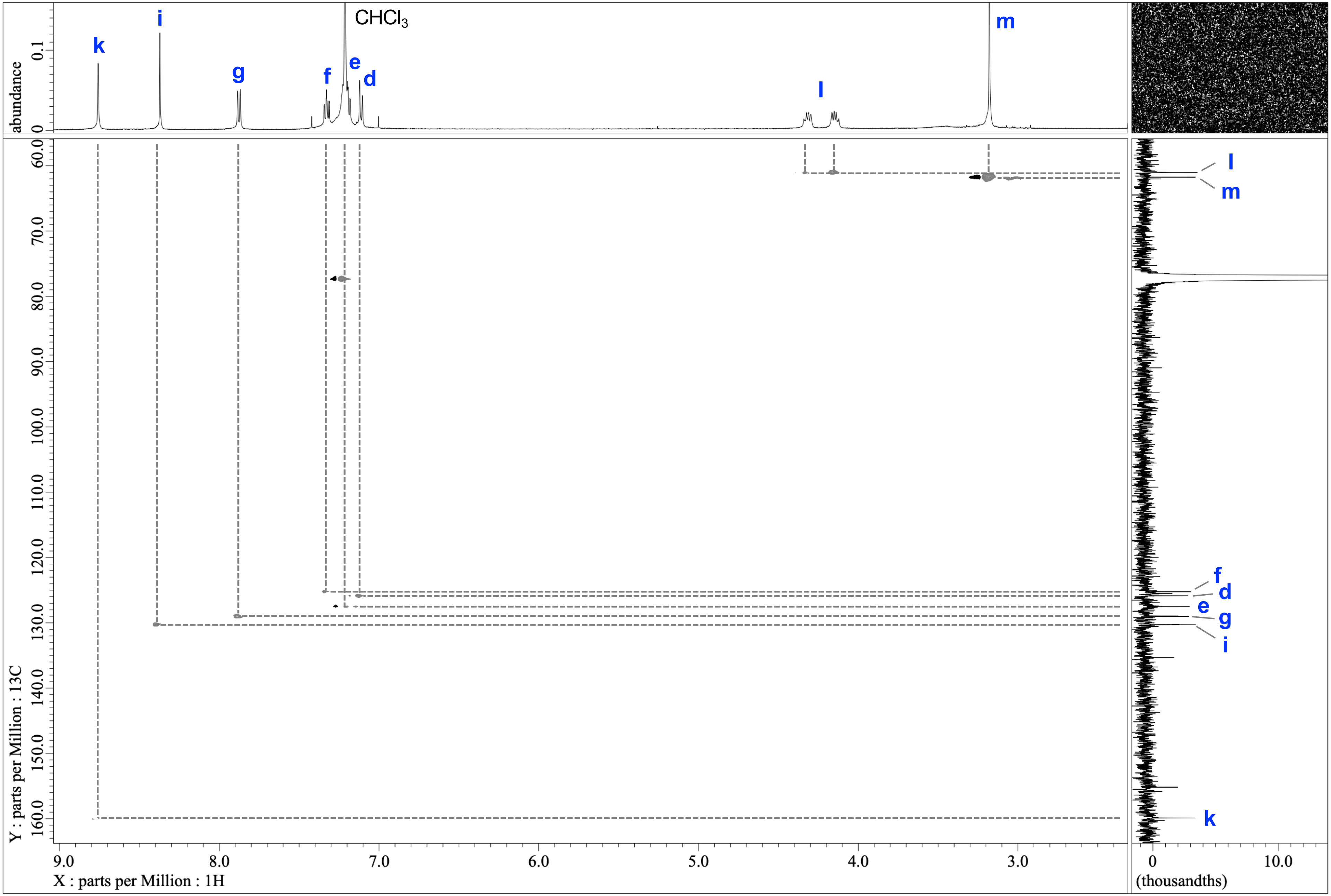

Partial HMBC NMR spectrum of (*S*,*R*)-**4** (500 MHz, $CDCl_3$, rt)

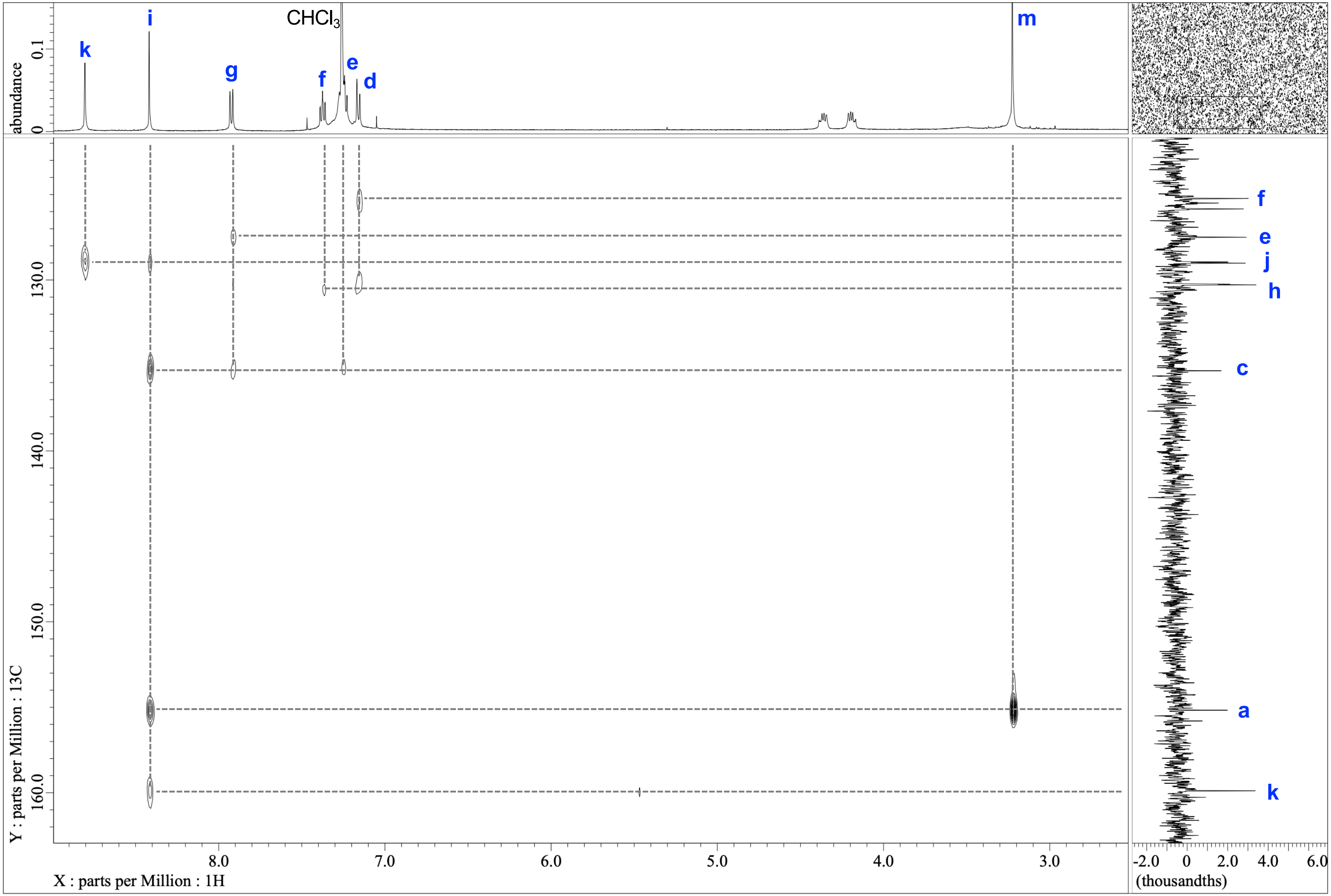

Supplement: Supplementary file 1 — ja4c01654_si_001.pdf [file ja4c01654_si_001.pdf]
